# Fbxo7 promotes Cdk6 activity to inhibit PFKP and glycolysis in T cells

**DOI:** 10.1083/jcb.202203095

**Published:** 2022-06-07

**Authors:** Rebecca Harris, Ming Yang, Christina Schmidt, Chloe Royet, Sarbjit Singh, Amarnath Natarajan, May Morris, Christian Frezza, Heike Laman

**Affiliations:** 1 Department of Pathology, University of Cambridge, Cambridge, UK; 2 Medical Research Council Cancer Unit, University of Cambridge, Cambridge Biomedical Campus, Cambridge, UK; 3 Pôle Chimie Balard Recherche, Institut des Biomolécules Max Mousseron-UMR5247, Montpellier, France; 4 Eppley Institute for Cancer Research, Fred & Pamela Buffett Cancer Center, University of Nebraska Medical Center, Omaha, NE

## Abstract

Fbxo7 is associated with cancer and Parkinson’s disease. Although Fbxo7 recruits substrates for SCF-type ubiquitin ligases, it also promotes Cdk6 activation in a ligase-independent fashion. We discovered PFKP, the gatekeeper of glycolysis, in a screen for Fbxo7 substrates. PFKP is an essential Cdk6 substrate in some T-ALL cells. We investigated the molecular relationship between Fbxo7, Cdk6, and PFKP, and the effect of Fbxo7 on T cell metabolism, viability, and activation. Fbxo7 promotes Cdk6-independent ubiquitination and Cdk6-dependent phosphorylation of PFKP. Importantly, Fbxo7-deficient cells have reduced Cdk6 activity, and hematopoietic and lymphocytic cells show high expression and significant dependency on Fbxo7. CD4^+^ T cells with reduced Fbxo7 show increased glycolysis, despite lower cell viability and activation levels. Metabolomic studies of activated CD4^+^ T cells confirm increased glycolytic flux in Fbxo7-deficient cells, alongside altered nucleotide biosynthesis and arginine metabolism. We show Fbxo7 expression is glucose-responsive at the mRNA and protein level and propose Fbxo7 inhibits PFKP and glycolysis via its activation of Cdk6.

## Teaser

Fbxo7 has dual roles, ubiquitining proteins and activating the kinase Cdk6. The authors show both Fbxo7 functions converge on phosphofructokinase (PFKP), a gatekeeper of glycolysis, inhibiting its function. Fbxo7 deficiency increases glycolysis and reduces T cell activation and viability, demonstrating its important role in regulating metabolism.

## Introduction

Fbxo7 (F-box protein only 7) is a clinically important protein implicated in a variety of pathologies, including anemia, cancer, and Parkinson’s disease (Nelson et al., 2013). Like other F-box proteins, Fbxo7 functions as a receptor for Skp1-Cullin1-F-box protein (SCF)–type E3 ubiquitin ligases; however, it also acts as scaffold for other multimeric proteins, notably the G1-phase cell cycle regulators Cdk6 and p27 (Nelson et al., 2013). Despite their high degree of homology, Fbxo7 selectively promotes Cdk6, but not Cdk4, binding to its activators, the D-type cyclins, increasing Cdk6 kinase activity (Laman et al., 2005). D-type cyclins and Cdk4/6 are core components of the cell cycle regulatory machinery which have in common the capacity to phosphorylate and inactivate the G1 checkpoint proteins of the retinoblastoma-associated protein family. The G1 phase cyclins and Cdks are often overexpressed in multiple cancers, including hematopoietic, breast, and brain tumors, and thus they are well-validated targets for inhibition by small molecules, such as palbociclib, ribociclib, and abemaciclib, where their inhibition ostensibly reduces cell cycle entry (Aleem and Arceci, 2015; Gao et al., 2020; Ingham and Schwartz, 2017). More recently, unique functions of Cdk6, distinct from Cdk4, in regulating specific transcription factors and cellular functions are being reported (Bellutti et al., 2018; Kollmann et al., 2016; Scheicher et al., 2015; Uras et al., 2017; Uras et al., 2016; Wang et al., 2017). This includes a pro-survival activity of cyclin D3/Cdk6 activity attributed to its phosphorylation and inhibition of glycolytic enzymes, phosphofructokinase (PFKP) and PKM2, in T acute lymphoblastic leukemia (T-ALL) cells and other tumors with high expression of Cdk6 (Wang et al., 2017).

Our studies to identify the binding partners and substrates of Fbxo7 revealed an overlapping set of candidates that were identified as cyclin D3/Cdk6 substrates, including PFKP, the prominent PFK1 isoform expressed in T-ALL cells (Anders et al., 2011; Stott et al., 2019; Teixeira et al., 2016). We investigated the molecular relationships between these three proteins, and the effect Fbxo7 has on T cell metabolism, viability, and activation. We found SCF^Fbxo7^ ubiquitinates PFKP; however, an interaction between Fbxo7 and PFKP does not require Cdk6. In contrast, Cdk6 interaction with PFKP is dependent on Fbxo7, and Fbxo7-deficient T cells have reduced Cdk6 activity. Cancer cell lines derived from blood and lymphocytic malignancies, which have high expression of Cdk6, show a significant dependency on Fbxo7. Both naive and activated CD4^+^ T cells isolated from Fbxo7-deficient mice show reduced cell viability and activation. Metabolomic studies of activated CD4^+^ T cells demonstrate that cells with reduced Fbxo7 have increased glycolytic flux compared to WT cells, alongside alterations in purine and pyrimidine biosynthesis and arginine metabolism. Our data show Fbxo7 is a dose-dependent, glucose-responsive gene. We propose Fbxo7 regulates metabolism at the entry of glucose into glycolysis by enabling Cdk6 phosphorylation of PFKP.

## Results

### PFKP is a substrate for ubiquitination by Fbxo7

**Figure 1.**
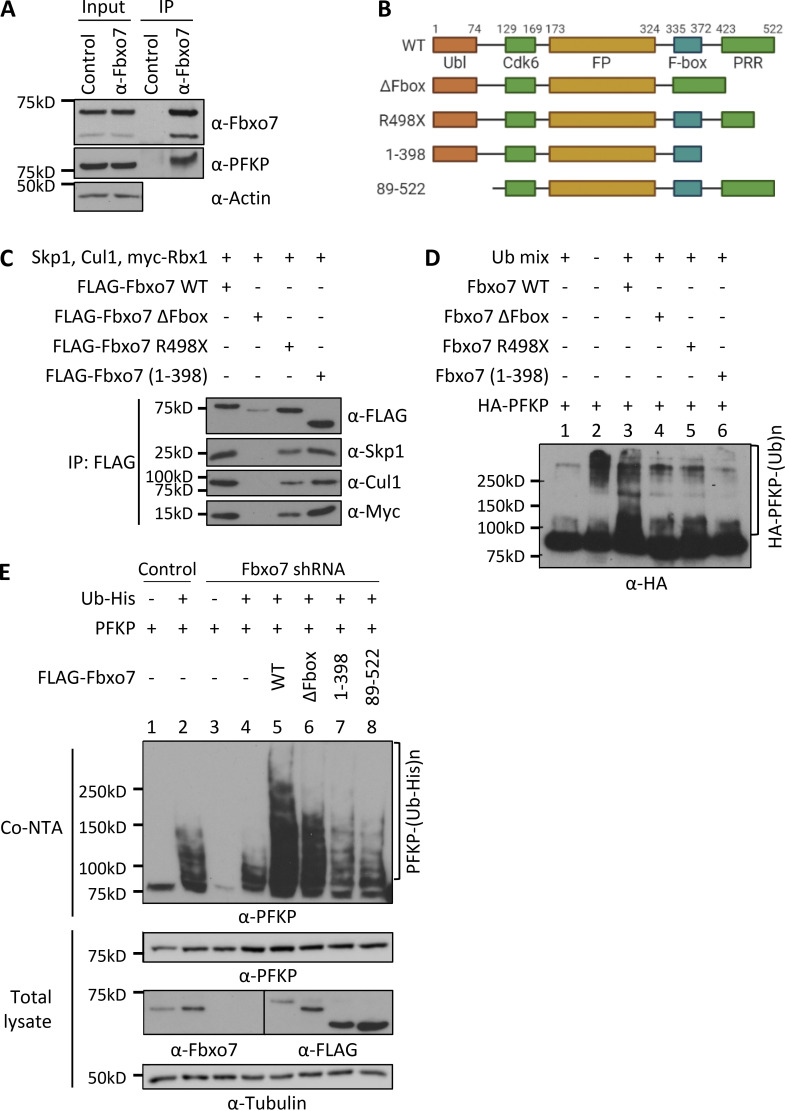
**Fbxo7 ubiquitinates PFKP. (A)** Fbxo7 immunoprecipitation (IP) from CCRF-CEM cells, showing co-immunoprecipitation of PFKP (*n* = 3). **(B)** Schematic of Fbxo7 constructs used to make SCF ligases for in vivo ubiquitination assays. All contain an N-terminal FLAG tag (not shown). Ubl, ubiquitin-like domain; Cdk6, Cdk6-binding domain; FP, Fbxo7-PI31 dimerization domain; PRR, proline rich region. **(C)** FLAG-Fbxo7 constructs were transfected into HEK293T cells alongside Skp1, Cullin1 (Cul1), and myc-Rbx1. SCF complexes were isolated by FLAG immunoprecipitation, and the presence of the other SCF components was confirmed by immunoblot. **(D)** In vitro ubiquitination assay of SCF^Fbxo7^ complexes in C, together with HA-PFKP and a ubiquitin mix (ubiquitin buffer, UBE1, UbcH5a, and ATP; *n* = 2). **(E)** In vivo ubiquitination assay of Fbxo7 constructs with PFKP. Constructs were overexpressed in HEK293T cells stably expressing a control or Fbxo7 shRNA. Ubiquitinated proteins were isolated using a cobalt-NTA affinity resin (Co-NTA) to the His-tag on ubiquitin. Immunoblot for PFKP shows the degree of ubiquitination following expression of each Fbxo7 construct (*n* = 3). Source data are available for this figure: SourceData F1.

To identify Fbxo7-interacting proteins, a yeast two-hybrid screen was performed using aa 334-522 of Fbxo7, which spans the F-box domain and the substrate-binding proline rich region, as bait. One candidate identified was aa 385−784 of 6-PFKP. To validate this interaction in mammalian cells, endogenous Fbxo7 was immunoprecipitated from the T-ALL cell line, CCRF-CEM. Both Fbxo7 isoforms 1 and 2 of 70 and 60 kD, respectively, were isolated and immunoblot analyses for PFKP confirmed a specific interaction between the endogenous proteins (Fig. 1 A).

Since F-box proteins recruit substrates to SCF-type E3 ligases, we next investigated whether SCF^Fbxo7^ ubiquitinated PFKP. Various FLAG-Fbxo7 constructs were transfected into HEK293T cells along with additional SCF components to generate WT and truncated Fbxo7 SCF E3 ubiquitin ligases, isolated by FLAG immunoprecipitation (Fig. 1, B and C). These E3 ligases were used for in vitro ubiquitination assays with HA-purified PFKP as a substrate (Fig. 1 D). Upon addition of SCF^Fbxo7^, we observed an increased smear of higher molecular weight bands representing poly-ubiquitinated PFKP (Fig. 1 D). This modification was dependent on the F-box domain (Fig. 1 D), which binds Skp1 and is required for SCF^Fbxo7^ complex formation (Fig. 1 C). Although two C-terminally truncated Fbxo7 proteins recruited SCF-ligase components (Fig. 1 C), these E3 ligases did not promote PFKP poly-ubiquitination (Fig. 1 D), indicating the C-terminus of Fbxo7, as used in the yeast two-hybrid screen, was required for PFKP ubiquitination.

We next performed in vivo ubiquitination assays in HEK293T cells expressing a control or Fbxo7-targeting shRNA. Cells were transfected with His-tagged ubiquitin, PFKP, and FLAG-Fbxo7 constructs, and ubiquitinated proteins were isolated by cobalt–nitrilotriacetic acid (NTA) affinity resin (Fig. 1 E). Immunoblot analyses showed a reduction in His-ubiquitinated PFKP in cells expressing shRNA to Fbxo7 compared to control shRNA (Fig. 1 E, lane 4 vs. lane 2). Re-expression of WT Fbxo7 markedly increased PFKP poly-ubiquitination (Fig. 1 E, lane 5), and this was reduced when the ΔF-box, N-terminally truncated, or C-terminally truncated Fbxo7 constructs were expressed (Fig. 1 E, lanes 6–8). The decrease, but not absence of ubiquitination evident in lane 6, may reflect multimer formation of the ΔF-box construct with residual WT Fbxo7. Together, these data show an interaction occurs between Fbxo7 and PFKP resulting in its ubiquitination by SCF^Fbxo7^, and this modification is dependent on both the N- and C-termini of Fbxo7.

### Fbxo7 promotes Cdk6-independent ubiquitination and Cdk6-dependent phosphorylation of PFKP

**Figure S1.**
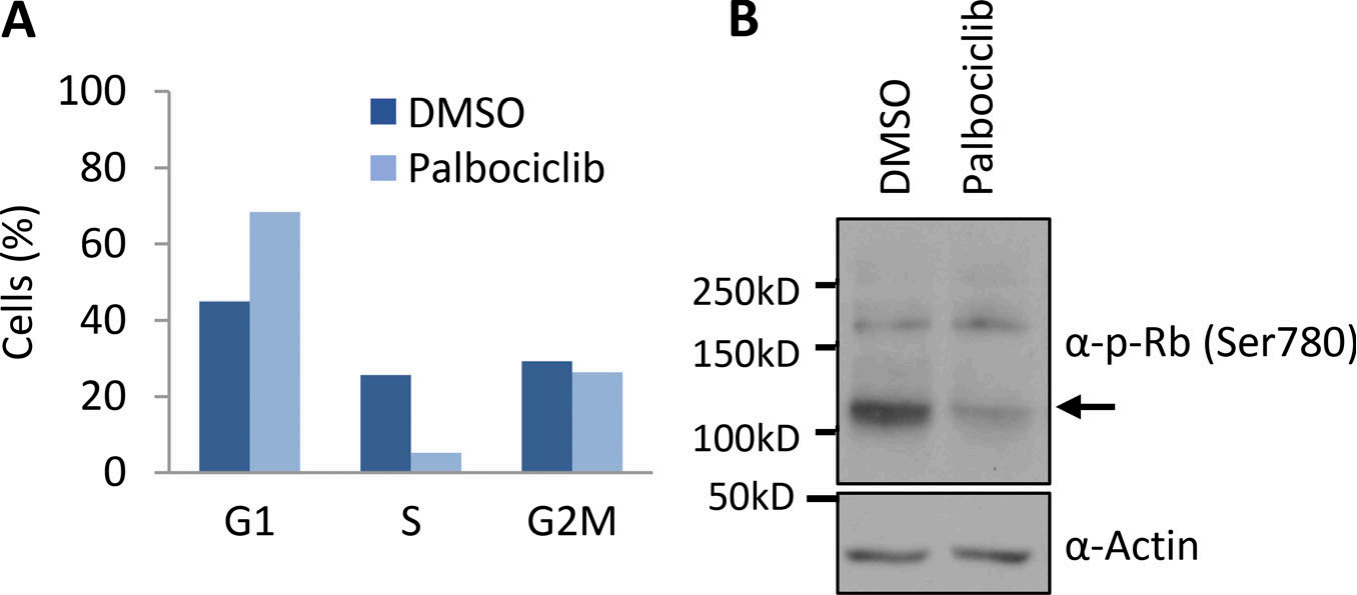
**Cells treated with palbociclib accumulate in G1 phase. (A)** Inhibition of Cdk4/6 by palbociclib in CCRF-CEM cells was verified by cell cycle analysis using PI staining (*n* = 2). **(B)** Inhibition of Cdk4/6 by palbociclib in HEK293T cells was verified by immunoblotting cell lysates for phospho-Rb at Ser780 (*n* = 1). Source data are available for this figure: SourceData FS1.

**Figure 2.**
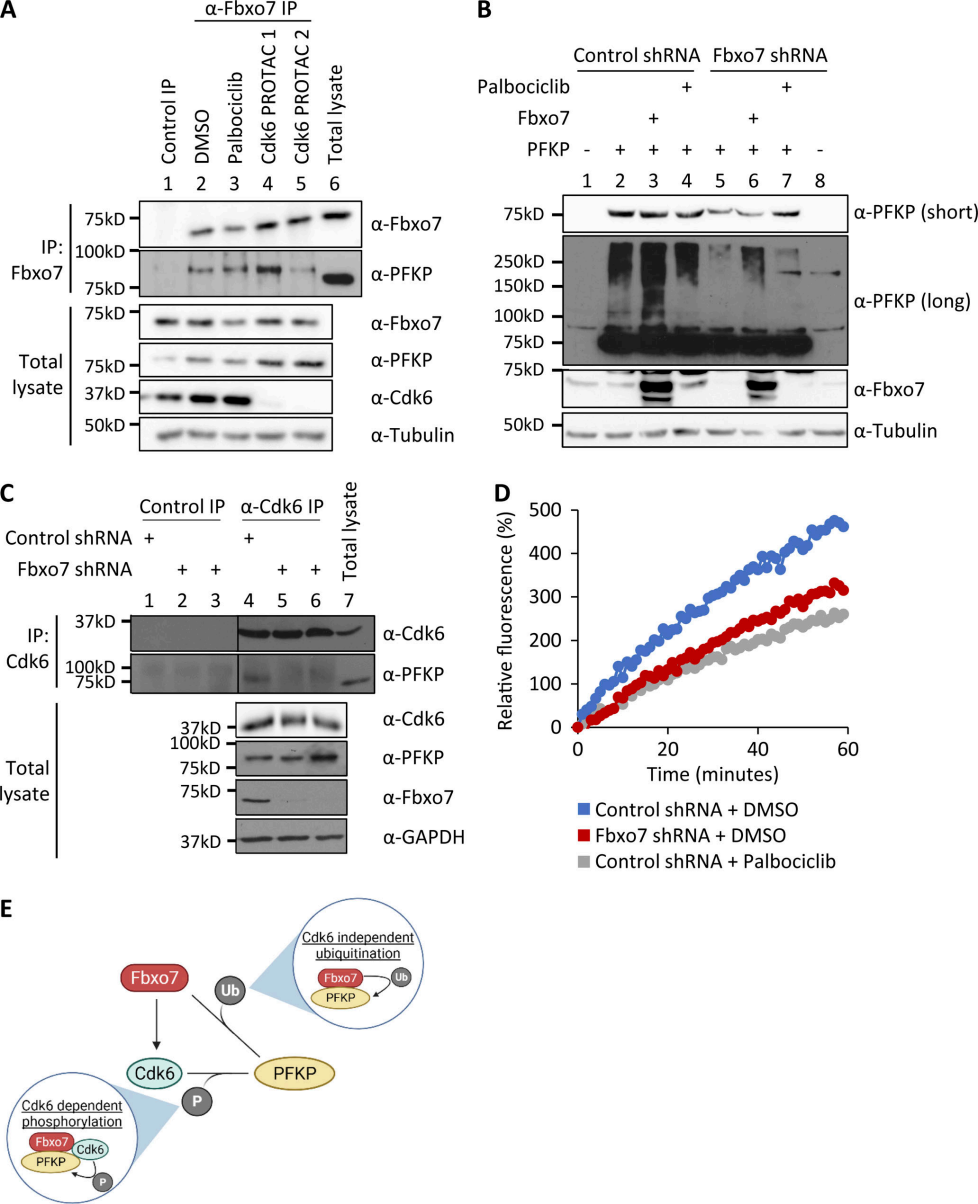
**Fbxo7 promotes the Cdk6-independent ubiquitination and Cdk6-dependent phosphorylation of PFKP. (A)** Fbxo7 immunoprecipitation from CCRF-CEM cells treated with 1 μM palbociclib or 0.1 μM of either of two different Cdk6 PROTACs for 24 h (Rana et al., 2019; Su et al., 2019). PFKP co-immunoprecipitation (IP) was analyzed by immunoblot (*n* = 2). **(B)** HEK293T cells with a control or Fbxo7 shRNA were transfected and treated with 1 μM palbociclib for 24 h as indicated. Cell lysates were resolved by SDS-PAGE and analysed by immunoblot (*n* = 2). **(C)** Cdk6 immunoprecipitation from CCRF-CEM cells expressing control or Fbxo7 shRNA. PFKP co-immunoprecipitation was analyzed by immunoblot (*n* = 1). **(D)** Cdk6 kinase activity in CCRF-CEM cells expressing control or Fbxo7 shRNA and treated with 1 μM palbociclib for 24 h as indicated. Kinase activity is measured in a dose-dependent manner by fluorescence of the Cdk6 biosensor (*n* = 1). **(E)** Diagram of model whereby Fbxo7 promotes both the ubiquitination (Ub) and phosphorylation (P) of PFKP. Source data are available for this figure: SourceData F2.

Cdk6 phosphorylates PFKP (Wang et al., 2017), and Fbxo7 binds directly to and specifically activates Cdk6 (Laman et al., 2005) and also ubiquitinates PFKP (Fig. 1, D and E). Since phospho-degrons are a common recognition motif for SCF E3 ligases, we investigated whether Cdk6 or its kinase activity was required for Fbxo7 to interact with PFKP. This was tested by treating cells with two different Cdk6-specific proteolysis targeting chimera (PROTAC) degraders (Rana et al., 2019; Su et al., 2019), or by treating cells with palbociclib. Inhibition of Cdk4/6 in palbociclib-treated cells was verified by immunoblotting cell lysates for phosphoSer780, a Cdk4/6 phospho-acceptor site in the retinoblastoma protein, and by profiling cell cycle parameters (Fig. S1). CCRF-CEM cells were treated with vehicle control (DMSO), 1 μM palbociclib, or 0.1 μM Cdk6 PROTAC for 24 h, prior to Fbxo7 immunoprecipitation (Fig. 2 A). PFKP co-immunoprecipitated with Fbxo7 in cells treated with palbociclib (Fig. 2 A, lane 3) and in Cdk6-PROTAC−treated cells (Fig. 2 A, lanes 4 and 5), demonstrating neither Cdk6 activity nor its presence was necessary for Fbxo7 binding to PFKP.

We tested if Cdk6 activity was required for SCF^Fbxo7^-mediated ubiquitination of PFKP. HEK293T cells expressing a control or Fbxo7-targeting shRNA were transfected with PFKP and treated with palbociclib. As seen previously, PFKP ubiquitination was reduced upon Fbxo7 knockdown (Fig. 2 B, lane 2 vs. lane 5) and enhanced with Fbxo7 overexpression (Fig. 2 B, lane 2 vs. lane 3). Moreover, 1 μM palbociclib treatment did not alter PFKP ubiquitination (Fig. 2 B, lane 2 vs. lane 4), indicating Cdk6 activity is not required for SCF^Fbxo7^ ubiquitination of PFKP.

**Figure S2.**
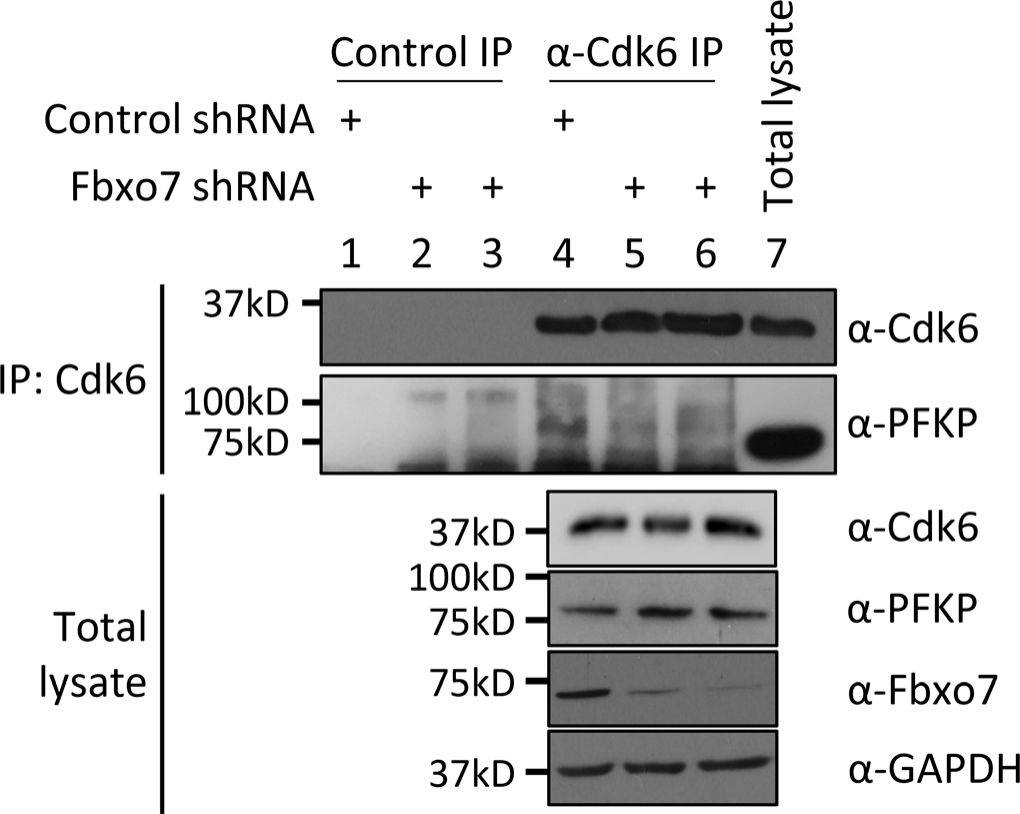
**Cdk6 immunoprecipitation (IP) from MOLT-4 cells expressing control or Fbxo7 shRNA.** PFKP co-immunoprecipitation was analyzed by immunoblot (*n* = 1). Source data are available for this figure: SourceData FS2.

Since Cdk6 was dispensable for Fbxo7 interaction and ubiquitination of PFKP, we tested whether Fbxo7 was required Cdk6 interaction with PFKP. Cdk6 was immunoprecipitated from CCRF-CEM cells expressing control or two different Fbxo7-targeting shRNAs (Fig. 2 C). Immunoblots show co-immunoprecipitation of PFKP with Cdk6 in the presence of Fbxo7 (Fig. 2 C, lane 4), but this interaction is lost upon Fbxo7 knockdown (Fig. 2 C, lanes 5 and 6). Similar results were obtained in MOLT-4 cells (Fig. S2). These data show Fbxo7 is required for a Cdk6-PFKP interaction and suggest Fbxo7 would be required for Cdk6 phosphorylation of PFKP. To test this, we utilised a fluorescent peptide biosensor for Cdk6 activity based on the Cdk6-phospho-acceptor in PFKP (Soamalala et al., 2021). Lysates from WT or Fbxo7-deficient CCRF-CEM cells were assayed using this biosensor (Fig. 2 D), and those with reduced Fbxo7 had lower Cdk6 activity, comparable to palbociclib-treated controls. These data show Fbxo7 is required for Cdk6 activity toward PFKP. Our data indicate Fbxo7 promotes two post-translational modifications, Cdk6 kinase-independent ubiquitination and Cdk6-dependent phosphorylation, on PFKP (Fig. 2 E).

### Knockdown of Fbxo7 reduces the pool of inactive monomer/dimer forms of PFKP

**Figure 3.**
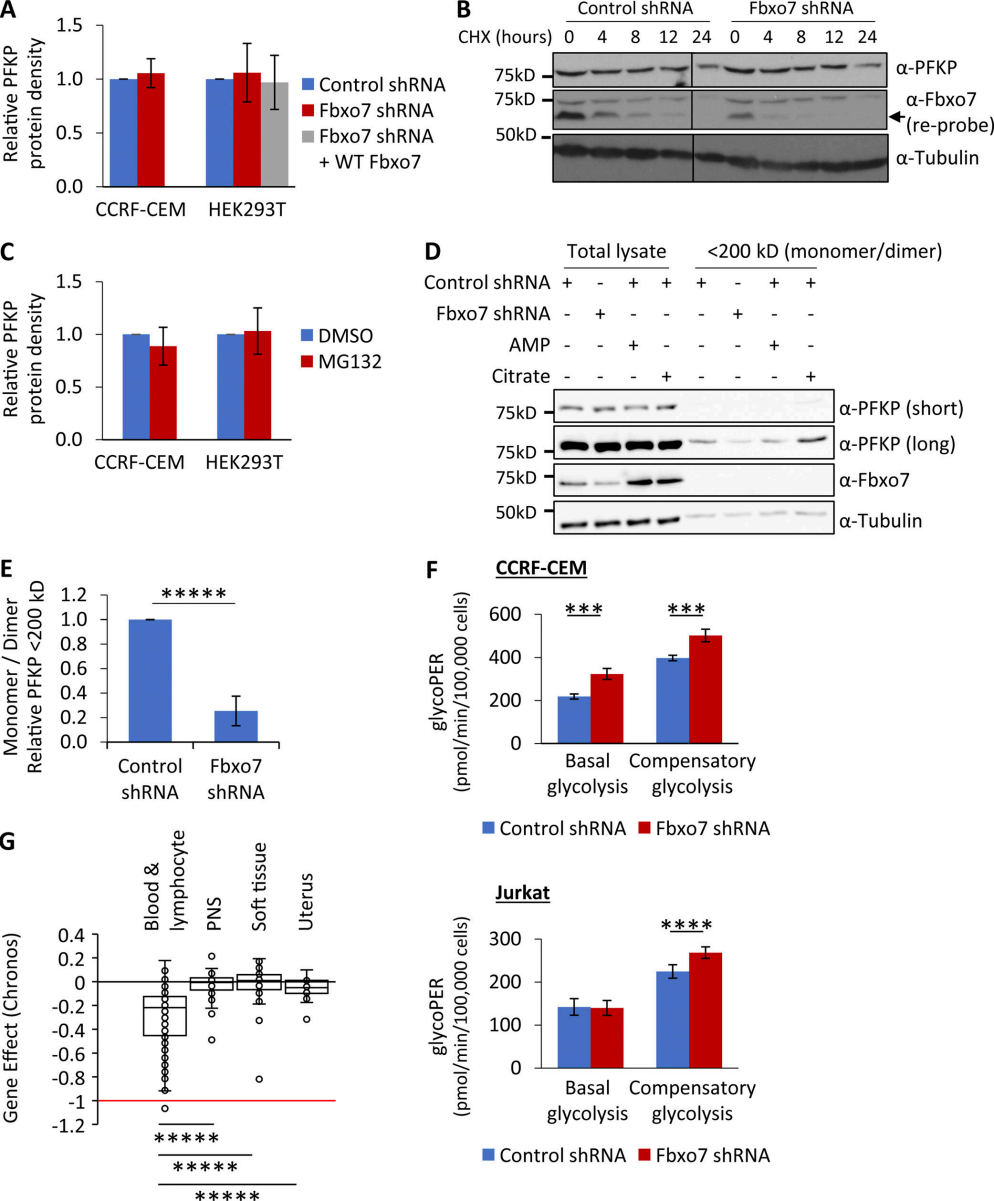
**Fbxo7 knockdown in T-ALL cells reduces the proportion of inactive monomer/dimer PFKP and promotes glycolysis. (A)** Lysates from cells expressing a control or Fbxo7 shRNA, and with Fbxo7 overexpression, were analyzed by immunoblot and quantified (CCRF-CEM *n* = 4, HEK293T *n* = 3). Data are presented as mean ± SD. **(B)** Image of representative immunoblot of PFKP half-life in CCRF-CEM cells expressing a control or Fbxo7 shRNA and treated with CHX for up to 24 h (*n* = 2). **(C)** Cells were treated with DMSO or 10 μM MG132 for 4 h and lysed. Proteins were resolved by SDS-PAGE to immunoblot for PFKP, and levels were quantified (CCRF-CEM *n* = 2, HEK293T *n* = 3). Data are presented as mean ± SD. **(D)** Lysates from CCRF-CEM cells with control or Fbxo7 shRNA were passed over a 200-kD molecular weight cutoff filter to separate PFKP monomers (86 kD) and dimers (172 kD) from tetramers (344 kD). AMP and citrate were added to lysates as controls to respectively promote or dissociate PFKP tetramers. Total lysates and <200 kD fractions were resolved by SDS-PAGE and analyzed by immunoblot (representative *n* = 4). **(E)** Quantification of PFKP in <200 kD fraction as in D (*n* = 4). Data are presented as mean ± SD. **(F)** Glucose metabolism in CCRF-CEM (top) and Jurkat E6 (bottom) cells expressing a control or Fbxo7 shRNA was analyzed by the Agilent Seahorse Glycolytic Rate Assay (*n* = 4). Basal glycolysis refers to the physiological rate, whereas compensatory glycolysis reflects the glycolytic capacity when mitochondrial respiration is inhibited. **(G)** Chronos gene dependency scores for *FBXO7* in cancer cell lines of various lineages, plotted using data publicly available on the DepMap portal (https://DepMap.org). A Chronos score of 0 indicates that a gene is nonessential, while −1 is comparable to the median of all pan-essential genes. Blood & lymphocyte *n* = 111, peripheral nervous system [PNS] *n* = 32, soft tissue *n* = 44, uterus *n* = 34. ***, P < 0.005; ****, P < 0.001; *****, P < 0.0005. Source data are available for this figure: SourceData F3.

One common outcome of ubiquitination is targeting to the 26S proteasome, so we tested whether Fbxo7 and PFKP protein levels correlated in CCRF-CEM and HEK293T cells. Immunoblot analyses of cell lysates showed no change in steady-state PFKP with either Fbxo7 knockdown or overexpression (Fig. 3 A). There was also no change in PFKP half-life in cells with reduced Fbxo7 levels (Fig. 3 B), and no accumulation of PFKP upon treatment with MG132 to inhibit the proteasome (Fig. 3 C). These data indicate that Fbxo7 does not affect PFKP steady state levels. However, a major mechanism for PFKP regulation is the formation and dissociation of tetrameric complexes. PFKP is most enzymatically active as a tetramer, but not as dimers/monomers. We therefore investigated whether Fbxo7 expression affects the distribution of PFKP complexes. Total cell lysates from CCRF-CEM cells expressing a shRNA targeting Fbxo7 or a nontargeting control were passed over an ultrafiltration unit with molecular mass 200 kD cutoff to separate monomers (86 kD) and dimers (172 kD) from active tetramers (344 kD). As controls, allosteric regulators of PFKP were added to total cell lysates prior to filtration to promote (AMP) or dissociate (citrate) PFKP tetramers. The filtrates (<200 kD fraction) containing the smaller dimer/monomers forms were then analyzed, alongside the lysates prior to filtration (Fig. 3 D). Immunoblots showed that Fbxo7 knockdown did not change total levels of PFKP, as before, but instead reduced the amount of inactive dimer/monomer forms by 75% (Fig. 3, D and E), suggesting a change in distribution of PFKP into active tetramers. We note that this phenotype is also observed when T-ALL cells were treated with palbociclib (Wang et al., 2017).

### Fbxo7 inhibits glycolysis in T-ALL cells, and hematopoietic and lymphoid cancer cells are dependent on Fbxo7

**Figure S3.**
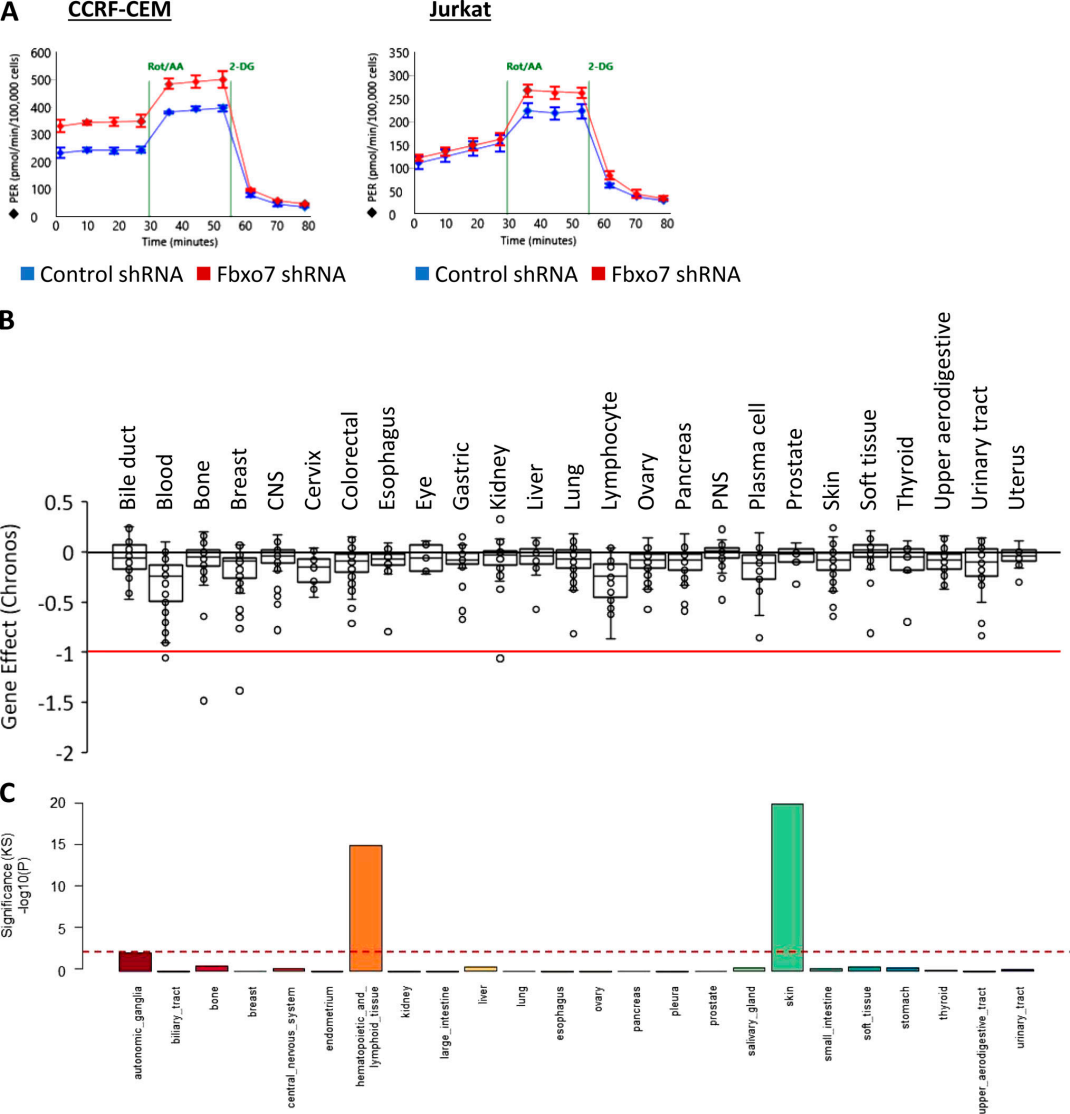
**Fbxo7 is highly expressed and required in hematopoietic and lymphoid cell lines. (A)** Representative Seahorse profiles measuring proton efflux rate (PER) over time for CCRF-CEM (left) and Jurkat E6 (right) cells expressing a control or Fbxo7 shRNA and using an Agilent Seahorse Glycolytic Rate Assay. **(B)** Box-and-whisker plot of Chronos gene dependency scores for *FBXO7* in 1,020 cancer cell lines of various lineage, plotted using data publicly available on the DepMap portal (https://DepMap.org). **(C)** A graph of one-sample Kolmogorov-Smirnov (KS) test of Fbxo7 expression in cell lines from CCLE, where dashed red line indicates a P value of 0.01. CNS, central nervous system; PNS, peripheral nervous system.

As our data suggested that Fbxo7 increases PFKP activity, we tested whether Fbxo7 affected glycolysis in cancer cell lines using an Agilent Seahorse to measure the glycolytic rate. We measured the glycolytic rate of T-ALL cells expressing a control or Fbxo7 shRNA. We found Fbxo7 knockdown increased both basal and compensatory glycolysis in CCRF-CEM cells, by 48 and 26%, respectively, while Jurkat E6 cells showed a 20% rise in compensatory glycolysis (Fig. 3 F and Fig. S3 A). These data indicate higher levels of glycolysis in malignant T cells with reduced Fbxo7. Consequently, in light of studies showing Cdk6 regulation of glycolytic enzymes is essential for the survival of T-ALL cells (Wang et al., 2017), we reasoned that Fbxo7 should be essential for viability in hematological malignancies, since Cdk6 is the major kinase expressed in these cell lineages. We assessed the requirement for Fbxo7 in over 1,000 cell lines by exploring the Cancer Dependency Map (Tsherniak et al., 2017) which provides a large-scale analysis of CRISPR-Cas9 screening data, and uses the Chronos algorithm to calculate a measure of gene knockout fitness (Dempster et al., 2021). Around 8% of cell lines were strongly selected as dependent on Fbxo7, and consistent with our model, we find that hematopoietic and lymphoid lineages are among the significantly enriched cell lines showing a preferential dependency on Fbxo7 (Fig. 3 G and Fig. S3 B). Fbxo7 is also highly expressed in these cell lineages (Fig. S3 C). These findings indicate an essential role for Fbxo7 in these malignancies.

### Metabolomic analysis on Fbxo7-deficient T cells shows broad metabolic alterations and increased glycolysis

**Figure 4.**
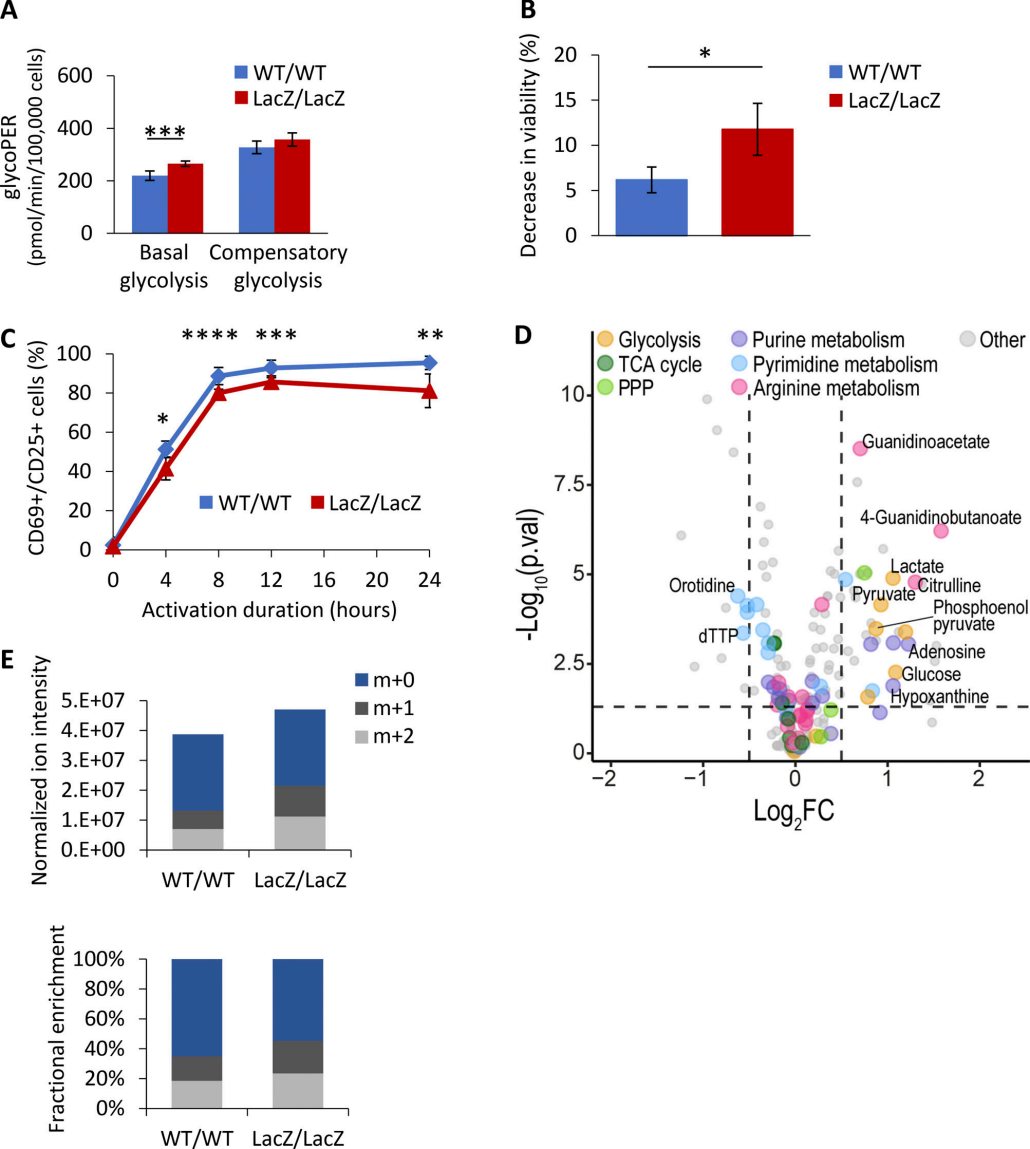
**CD4**^**+**^ **T-cells from mice lacking Fbxo7 display metabolic reprogramming and enhanced glycolysis, alongside survival and activation defects. (A)** CD4^+^ T cells from WT or Fbxo7^LacZ/LacZ^ mice were activated for 48 h, then their glucose metabolism was analyzed by the Agilent Seahorse Glycolytic Rate Assay (*n* = 2). Basal glycolysis refers to the physiological rate, whereas compensatory glycolysis reflects the glycolytic capacity when mitochondrial respiration is inhibited. **(B)** Decrease in viability of CD4^+^ T cells over 24 h of activation in vitro (*n* = 3). **(C)** Activation of CD4^+^ T cells in vitro, as measured by CD69 and CD25 expression over 24 h (*n* ≥ 3). **(D)** Volcano plot of differentially expressed metabolites from untargeted metabolomics profiling of CD4^+^ T cells from WT or Fbxo7^LacZ/LacZ^ mice, activated for 48 h (*n* = 6). **(E)** CD4^+^ T cells from WT or Fbxo7^LacZ/LacZ^ mice were activated for 24 h in the presence of glucose-1,2-^13^C_2_, then subjected to metabolomics analysis (*n* = 6). m+1 and m+2 intracellular lactates are derived from radio-labeled glucose. Data are presented as mean ± SD; *, P < 0.05; **, P < 0.01; ***, P < 0.005; ****, P < 0.001.

**Figure S4.**
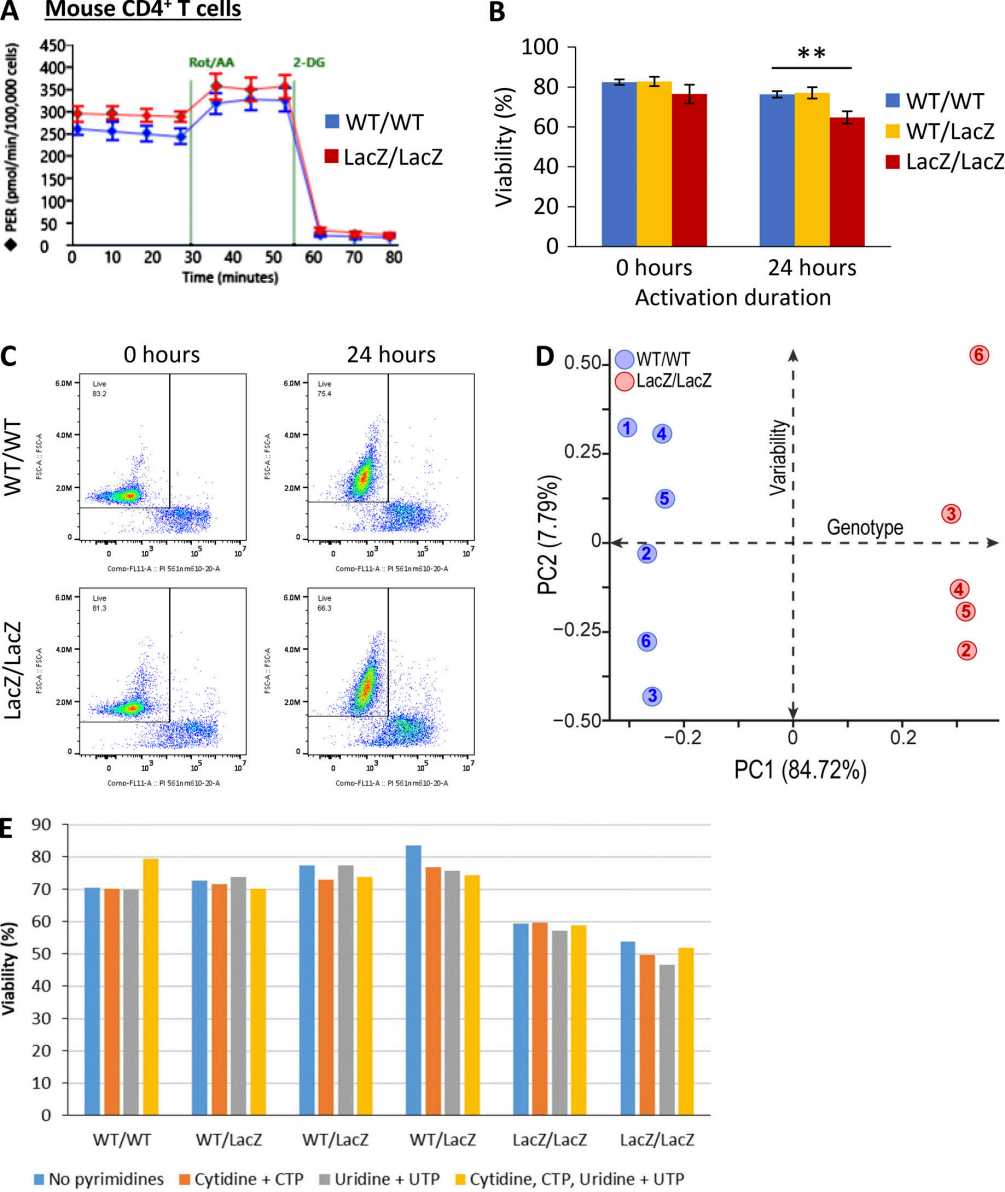
**Fbxo7 is required for primary CD4^+^ T cell viability and activation. (A)** Representative Seahorse profiles for CD4^+^ T cells from WT or Fbxo7^LacZ/LacZ^ mice activated for 48 h and analyzed by Agilent Seahorse Glycolytic Rate Assay. **(B and C)** Viability of CD4^+^ T cells upon isolation and 24 h after activation in vitro (*n* = 3). **(B)** Summary quantification. **(C)** Example FACS plots of PI staining. **(D)** PCA of intracellular metabolite ion intensities obtained from untargeted metabolomics profiling of CD4^+^ T cells from WT or Fbxo7^LacZ/LacZ^ mice, activated for 48 h (*n* = 6). **(E)** Viability of CD4^+^ T cells isolated from WT, Fbxo7^+/LacZ^, or Fbxo7^LacZ/LacZ^ mice after 24 h of in vitro activation in the presence of exogenous pyrimidines, as indicated. CTP, cytidine triphosphate; UTP, uridine triphosphate.

To determine if Fbxo7 also increases glycolysis in primary T cells, where glycolysis is upregulated upon activation, we tested the effect of Fbxo7 in T cells from WT or an Fbxo7-deficient mouse, in which the *Fbxo7* locus is disrupted by a *LacZ* insertion (Ballesteros Reviriego et al., 2019; Meziane et al., 2011; Randle et al., 2015). WT and mutant splenic CD4^+^ T-cells were isolated and activated in vitro for 48 h prior to analysis (Fig. 4 A and Fig. S4 A). Fbxo7-deficient T cells exhibited significantly higher basal glycolysis and a smaller increase in compensatory glycolysis (Fig. 4 A and Fig. S4 A). In addition, we noted after activation, the viability of mutant T cells was significantly reduced by 1.9-fold compared to WT cells (Fig. 4 B and Fig. S4, B and C), and the activation of Fbxo7-deficient cells was delayed and plateaued at 14.2% lower levels than WT (Fig. 4 C). Thus, despite decreased viability and lower levels of activation, Fbxo7-deficient CD4^+^ T cells showed increased glycolysis compared to WT cells.

To explore the impact of Fbxo7 on metabolism, we performed untargeted metabolomics profiling on activated splenic CD4^+^ T cells. Principal component analysis shows discrete clusters of WT and mutant samples, illustrating divergent metabolic profiles (Fig. S4 D). Variations in the T cells from mutant Fbxo7^LacZ/LacZ^ mice were multifaceted and included a dysregulation in arginine metabolism and a nucleotide imbalance caused by an increase in purines and reduction of pyrimidines (Fig. 4 D). We considered whether the reduced pyrimidines might cause the reduced viability of T cells upon activation; however, supplementation of the media with pyrimidines did not rescue the mutant T cells (Fig. 4 E). Importantly, there is a pronounced accumulation of lactate, pyruvate, and late-stage glycolytic intermediates in the Fbxo7-deficient T cells (Fig. 4 D), supporting our hypothesis that glycolysis is altered in these cells.

To attribute this accumulation of glycolytic intermediates to increased glycolytic flux, we completed a stable isotope tracing study with CD4^+^ T-cells activated for 24 h in the presence of Glucose-1,2-^13^C_2_. There was a 1.68-fold increase in m+1 and 1.59-fold increase in m+2 labeled lactate in T cells from Fbxo7-deficient mice compared to WT (Fig. 4 E), confirming an increase in lactate formation via glycolysis. These data reveal the broad metabolic alterations caused by a loss of Fbxo7 in CD4^+^ T cells and, crucially, highlight an increase in glycolytic flux, indicating Fbxo7 is a negative regulator of glycolysis in primary T cells. Overall, these data indicate higher levels of glycolysis in primary and malignant T cells with reduced Fbxo7, demonstrating Fbxo7 negatively regulates glycolysis.

### Glucose starvation induces Fbxo7 degradation by autophagy

**Figure 5.**
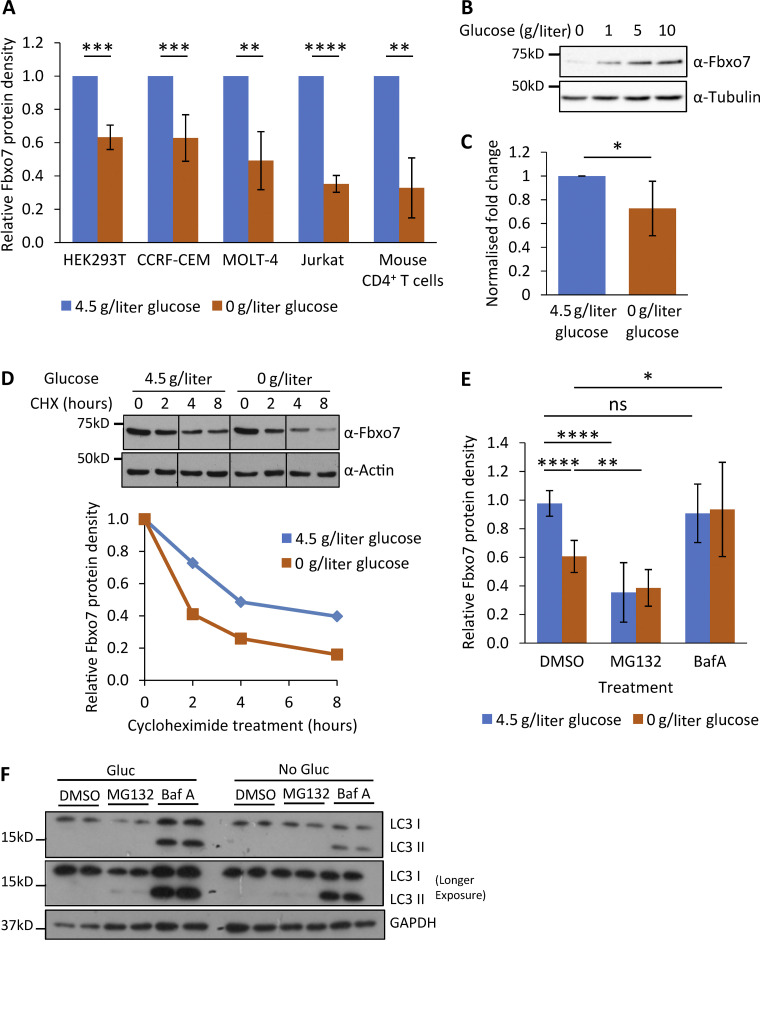
**Regulation of Fbxo7 by glucose availability. (A)** Relative Fbxo7 protein following 48 h of glucose starvation, analyzed by immunoblot and quantified (*n* ≥ 3). **(B)** CCRF-CEM cells cultured for 48 h with a titration of glucose, then lysed and analyzed by immunoblot (*n* = 3). **(C)** Relative Fbxo7 mRNA in naive murine CD4^+^ T cells, incubated for 4 h in 0 or 4.5 g/liter glucose (*n* = 6). **(D)** CHX chase in CCRF-CEM cells following the removal (0 g/liter) or maintenance (4.5 g/liter) of glucose. Fbxo7 protein measured by immunoblot (top) and quantified (bottom; representative of *n* = 3). **(E)** CCRF-CEM cells were treated with DMSO, 10 μM MG132, or 200 nM BafA1 (BafA) immediately following glucose removal (0 g/liter) or maintenance (4.5 g/liter). Fbxo7 protein measured by immunoblot and quantified (*n* = 7). **(F)** Image of representative immunoblot for LC3I/II from samples from E (*n* = 4). *, P < 0.05; **, P < 0.01; ***, P < 0.001; ****, P < 0.0001. Source data are available for this figure: SourceData F5.

The expression of glycolytic proteins is often responsive to glucose levels, so we tested whether Fbxo7 expression is influenced by glucose availability. HEK293T and three T-ALL cell lines were cultured in 4.5 or 0 g/liter glucose for 48 h prior to lysis and immunoblotting. Fbxo7 protein was reduced by up to 65% following glucose starvation (Fig. 5 A). Likewise, primary mouse CD4^+^ T cells activated for 48 h in the absence of glucose showed a 67% decrease in Fbxo7 (Fig. 5 A). Furthermore, there was a dose-dependent effect of glucose on Fbxo7 expression even up to hyperglycemic levels (10 g/liter; Fig. 5 B). The reduction in Fbxo7 expression upon glucose withdrawal correlated with a 25% decrease in mRNA levels (Fig. 5 C), and a shorter protein half-life as seen in a time course of cycloheximide (CHX) treatment of CCRF-CEM cells (*t*_*½*_ = 4 h in 4.5 g/liter glucose vs. *t*_½_ = 1.75 h in no glucose; Fig. 5 D). To determine the pathway promoting Fbxo7 degradation upon glucose removal, CCRF-CEM cells were treated with 10 μM MG132 or 200 nM bafilomycin A (BafA1) to inhibit the proteasome or autophagy, respectively (Fig. 5 E). Surprisingly, proteasome inhibition with MG132 downregulated Fbxo7, but this was not dependent on glucose concentration. BafA1 treatment rescued Fbxo7 levels after glucose withdrawal, suggesting Fbxo7 was cleared by autophagy. Immunoblotting for LC3I/II showed that irrespective of glucose levels, BafA1 treatment increased the levels of LC3II, consistent with the inhibition of autophagy, while MG132 treatment caused a subtle increase in LC3II (Fig. 5 F). These data show Fbxo7 expression correlates with glucose levels, and its regulation occurs both transcriptionally and post-translationally.

## Discussion

We show here Fbxo7 promotes two types of post-translational modifications on PFKP whereby Fbxo7 promotes Cdk6 kinase activity and can also independently ubiquitinate PFKP. We observed several phenotypes when Fbxo7 is reduced in primary and malignant T cells, including decreases in the inactive dimer/monomer forms of PFKP, increases in glycolysis, and reduced T cell activation phenocopying the loss or inhibition of Cdk6 activity (Hu et al., 2009; Hu et al., 2011; Malumbres et al., 2004; Wang et al., 2017). This supports the idea that a major role for Fbxo7 is Cdk6 activation, and our findings using a biosensor based on a Cdk6 phospho-acceptor in PFKP support this idea. Although PFKP was clearly ubiquitinated by SCF^Fbxo7^ independent of Cdk6 activity, reducing Fbxo7 did not alter the steady state levels of PFKP. However, other ubiquitin ligases, TRIM21 and A20, have been reported to catalyse ubiquitination-mediated degradation of PFKP in glioblastoma and PFKL in hepatocellular carcinoma (Feng et al., 2020; Lee et al., 2017). Although the nondegradative effects of Fbxo7-mediated ubiquitination of PFKP in T cells remain to be elucidated, we hypothesize an essential role for Fbxo7 in cancer cell lines in blood lineages is as an activator of Cdk6. In support of this, we found that hematological and lymphocytic malignancies, where Cdk6 is highly expressed, also have high expression of Fbxo7 and are specifically dependent on Fbxo7. Interestingly, we noted in our studies it was not possible to overexpress Fbxo7 in T cell lines, despite multiple attempts with various transfection and transduction strategies, suggesting both minimum and maximal levels of Fbxo7 expression in T cells.

Quiescent T cells predominantly use oxidative phosphorylation for their energy needs, while activated T cells increase glycolysis and glutaminolysis, alongside reactive oxygen species production. Moreover, differentiated T cell subsets display discrete metabolic profiles, with effector T cells notably more glycolytic than regulatory and memory subsets. Interestingly, we show that activated Fbxo7-deficient CD4^+^ T cells have higher levels of glycolysis despite significantly lower levels of viability and activation, and an increase in the proportion of less glycolytic regulatory and central memory T cell subsets in mutant mice (Lomonosov et al., 2011; Patel et al., 2016). Our analysis of the metabolome of activated CD4^+^ T cells lacking Fbxo7 also indicates that, in addition to increases in glycolysis, these primary cells have numerous metabolic alterations, including in arginine metabolism and in purine and pyrimidine biosynthesis. The central role of arginine in T cell metabolism makes this an interesting area for future investigation (Geiger et al., 2016). T leukemic cancer cell lines show perturbed glucose metabolism, as demonstrated by the Wang et al. (2017), study showing the reliance of T-ALL cells on shunting glucose into the pentose phosphate pathway to buffer against excessive reactive oxygen species. As we have demonstrated that hematopoietic and blood cancers are dependent on Fbxo7 expression, future studies on Fbxo7-regulated metabolic pathways in T cell malignancies may reveal actionable vulnerabilities.

Fbxo7 is a widely expressed protein, most highly in blood and testes. Fbxo7 misregulation and mutation are associated with a variety of pathological conditions, from anemia, male sterility, cancer, and Parkinson’s disease, demonstrating its critical role in multiple cell types. The diversity of these cell types raises the question of which Fbxo7-regulated pathways underlying cellular dysfunction are common or cell type–specific. We demonstrate that Fbxo7 expression is responsive to glucose levels at both the mRNA and protein levels, suggesting Fbxo7 levels may act to adjust the rate of glycolysis under different physiological and pathological conditions. Moreover, our data are consistent with a model where the major pathway for decreasing Fbxo7 levels under low glucose conditions in CEM cells is autophagy. This suggests that autophagic regulation of Fbxo7 levels under low glucose conditions would also feedback on the rate of glycolysis. Ubiquitin ligases are generally increased upon proteasome inhibition, but surprisingly, we observed lowered Fbxo7 protein levels under both high and low glucose conditions with MG132 treatment. One possibility is that proteasome inhibition activates a pathway that ultimately targets Fbxo7 for degradation, but only a subtle increase in LC3II is seen upon proteasome inhibition, suggesting autophagy was not greatly enhanced upon MG132 treatment. An alternate possibility is that Fbxo7 has recently been shown to bind and activate a degradative caspase pathway in neuroblastoma and HEK293T cells (Lee et al., 2021). Whether in specialized cell types, variations in glucose concentrations engage different degradative pathways for Fbxo7 with differential effects on glucose metabolism is an area for future investigation. Overall, our data indicate that Fbxo7 is degraded by an autophagic pathway in CEM cells.

We propose Cdk6 activity will respond to fluctuating glucose levels and potentially other stress conditions through the fine-tuning of Fbxo7 levels (Zhou et al., 2016). In addition to regulating metabolism, another critical role for Cdk6 under stress conditions has been attributed to its regulation of transcription (Bellutti et al., 2018; Handschick et al., 2014). Bellutti et al. (2018) proposed that Cdk6 antagonizes p53-mediated stress responses arising in bone marrow cells upon oncogenic stress, and in the absence of Cdk6, the pro-apoptotic functions of p53 prevail. In a Parkinson’s disease mouse model with conditional loss of Fbxo7 in dopaminergic neurons, a p53 pro-apoptotic signature was detected (Stott et al., 2019). One possibility is the progressive neuronal death observed in this mouse arises from a lack of Fbxo7 and consequently Cdk6, to offset the p53 pro-apoptotic transcriptional response. Although Fbxo7 impacts on several other cellular pathways, as a factor that selectively scaffolds Cdk6, Fbxo7 levels may help to set a threshold for Cdk6-directed stress responses, transcriptional and metabolic, in many different cell types. Future efforts will be aimed at understanding whether the regulation of Cdk6 represents a common, therapeutically tractable, pathway mediating Fbxo7’s effects in physiological and pathological settings.

## Materials and methods

### Experimental model details

#### Mice

All experiments in mice were performed in accordance with the UK Animals (Scientific Procedures) Act 1986 and Animal Research: Reporting of In Vivo Experiments guidelines. Animal licenses were approved by the Home Office and the University of Cambridge’s Animal Welfare & Ethical Review Body Standing Committee. Experiments were performed under Home Office license PPL 70/9001. Fbxo7^LacZ^ mice (Fbxo7^tm1a (EUCOMM) Hmgu^ on a C57BL/6J background) were bred as heterozygous crosses. WT, heterozygous, and homozygous littermates were harvested between 6 and 9 wk. Where littermates were not available, mice of a similar age were compared. Both female and male mice were used for experiments.

#### Cell lines

HEK923T cells and the human T-ALL cell lines CCRF-CEM, MOLT-4, and Jurkat E6 were purchased from American Type Culture Collection. Cells were maintained in DMEM (HEK293T) or RPMI (T-ALL cells) supplemented with 10% heat-inactivated fetal bovine serum (Gibco) and 100 U/ml penicillin and streptomycin (Gibco) at 37°C in a humidified 5% CO_2_ atmosphere. Cell lines stably expressing shRNA to human *FBXO7* were generated as described previously (Burchell et al., 2013; Meziane et al., 2011; Randle et al., 2015). Briefly, human Fbxo7sh1: 5′-CGC​CCA​GTC​TGG​TGT​TTG​GAA​T-3′ (HP_3087), or human Fbxo7sh2: 5′-CGC​TGA​GTC​AAT​TCA​AGA​TAA​T-3′ (HP_434828) were subcloned into MSCV-LTRmiR30-PIG (LMP) vectors, and retroviruses were used to infect target cells. Cells are sorted for GFP and then cloned by limiting dilution with selection in puromycin 2 μg/ml (Sigma-Aldrich). To vary glucose concentration, cells were cultured in DMEM or RPMI without glucose, and glucose solution (Thermo Fisher Scientific) was supplemented as required.

### Method details

#### Antibodies

Antibodies against the following proteins were used for immunoblotting: rabbit antihuman Fbxo7 (generated in Laman et al. [2005]), rabbit antihuman PFKP (CST 8164 or ab233109; Abcam), rabbit antihuman Cdk6 (sc-177; Santa Cruz), mouse antihuman Skp1 (610530; BD Biosciences), rabbit antihuman Cullin1 (sc-11384; Santa Cruz), rabbit anti-Myc-epitope tag (CST 2272), mouse anti-FLAG-epitope-tag (F3165; Sigma-Aldrich), rabbit anti-Actin (A2066; Sigma-Aldrich), mouse anti-Tubulin (T6557; Sigma-Aldrich), rabbit anti-GAPDH (G9545; Sigma-Aldrich), and rabbit antihuman Phospho-Rb (Ser780; CST 9307). Signal detection was by ECL (GE Healthcare) or SuperSignal West Pico PLUS Chemiluminescent Substrate (Thermo Fisher Scientific).

#### DNA constructs

pSG5-HA-PFKP was kindly provided by Kyung-Sup Kim (Department of Biochemistry and Molecular Biology, Yonsei University, Seoul, South Korea) and subcloned into pcDNA3. pcDNA3 vectors expressing full-length, truncated, or ΔF-box Fbxo7 with an N-terminal Flag tag have been previously described (Kirk et al., 2008).

#### Immunoprecipitation

For immunoprecipitation, cells were lysed in Tween Lysis Buffer (50 mM Hepes, 150 mM NaCl, 0.1% Tween-20, 10% Glycerol, 1 mM EDTA, 2.5 mM EGTA, 1 mM DTT, 10 mM β-glycerophosphate) with a protease inhibitor cocktail (Sigma-Aldrich) and other inhibitors (1 mM PMSF, 10 mM NaF, 1 mM Na_3_VO_4_) and disrupted with mild sonication. Lysates were incubated with 0.4 μg rabbit antihuman Cdk6 (sc-177; Santa Cruz) or 1 μg rabbit antihuman Fbxo7 (ARP43128_P050; Aviva Systems Biology) antibodies, or isotype matched control, for 1 h at 4°C with rotation, then 20 μl Protein A/G PLUS-Agarose (Santa Cruz) was added and samples were incubated for a further 2 h. Beads were washed four times in lysis buffer and resuspended in 2XLaemmli loading buffer.

#### Purification of SCF^Fbxo7^ complexes and substrates

HEK293T cells were transfected with Skp1, Cullin1, and Myc-Rbx1, alongside FLAG-Fbxo7 constructs. After 48 h, cells were resuspended in lysis buffer (50 mM Tris-HCl, pH 7.5, 225 mM KCl, and 1% NP-40) with a protease inhibitor cocktail (Sigma-Aldrich) and other inhibitors (1 mM PMSF, 10 mM NaF, 1 mM Na_3_VO_4_). Lysates were incubated with Anti-FLAG M2 Affinity Gel (Sigma-Aldrich) for 4 h at 4°C with rotation. Beads were washed three times in lysis buffer and twice in elution buffer (10 mM Hepes, 225 mM KCl, 1.5 mM MgCl_2_, 0.1% NP-40), and protein was eluted with 100 µg/ml FLAG peptide (Sigma-Aldrich) in elution buffer for 1 h at 4°C with rotation. Purified SCF complexes were stored at −20°C in 15% glycerol.

#### In vitro ubiquitination assays

To purify substrate for in vitro ubiquitination, HEK293T cells were transfected with HA-PFKP, which was immunoprecipitated with mouse anti-HA agarose (Sigma-Aldrich) 48 h after transfection. Substrate was eluted with 300 µg/ml HA tag peptide (Sigma-Aldrich) and stored at 20°C in 15% glycerol. For the in vitro ubiquitination assay, a ubiquitin mix was prepared with 100 nM E1 (UBE1; Bio-Techne), 500 nM E2 (UbcH5a; Bio-Techne), 20 µM human recombinant ubiquitin (Santa Cruz), and 2 mM ATP (Bio-Techne) in 1× ubiquitin conjugation reaction buffer (Bio-Techne). This was incubated for 5 min at RT, then added to 100 nM SCF and 1 μl HA-PFKP substrate and incubated for 1 h at 30°C. The entire 10-μl reaction was mixed with an equal volume of 2XLaemmli loading buffer, resolved by SDS-PAGE and analyzed by immunoblotting.

#### In vivo ubiquitination assays

HEK293T cells were transfected with His-tagged ubiquitin, PFKP, and FLAG-Fbxo7 constructs, and cells were treated with 25 μM MG132 for 4 h prior to lysis. Cells were harvested and a 10% portion was lysed in RIPA buffer (50 mM Tris, pH 7.5, 150 mM NaCl, 1% NP-40, 0.5% sodium deoxycholate, 0.1% SDS) for the total lysate sample. The remaining cells were resuspended in CoNTA lysis buffer (6 M guanidinium-HCl, 100 mM Na_2_HPO_4_/NaH_2_PO_4_, 10 mM Tris-HCl, pH 8, 5 mM imidazole, 10 mM β-mercaptoethanol) with 5 mM *N*-ethylmaleimide (Sigma-Aldrich), protease inhibitor cocktail (Sigma-Aldrich), and other inhibitors (1 mM PMSF, 10 mM NaF, 1 mM Na_3_VO_4_), and disrupted with mild sonication. Lysates were incubated with Super Cobalt NTA Agarose Affinity Resin (Generon) for 4 h at 4°C with rotation. Beads were washed once in CoNTA lysis buffer, once in CoNTA wash buffer (8 M urea, 100 mM Na_2_HPO_4_/NaH_2_PO_4_, 10 mM Tris-HCl, pH 6.8, 5 mM imidazole, 10 mM β-mercaptoethanol), and twice in CoNTA wash buffer containing 0.1% Triton-X 100, with an incubation of 5 min at RT between each wash. Beads were incubated in CoNTA elution buffer (200 mM imidazole, 150 mM Tris-HCl, pH 6.8, 30% glycerol, 5% SDS, 720 mM β-mercaptoethanol) for 20 min at RT with shaking to elute His-tagged proteins. Both total lysate and eluate were resolved by SDS-PAGE and analyzed by immunoblotting.

#### Drug treatments

Where indicated, cells were treated with 10 μM MG132 (Sigma-Aldrich), 200 nM bafilomycin A1 (Santa Cruz Biotechnology), 100 µg/ml cycloheximide (Sigma-Aldrich), 1 µM palbociclib (Sigma-Aldrich), or 0.1 µM Cdk6 PROTAC for the indicated durations. Both Cdk6 PROTACs utilise palbociclib as the ligand for Cdk6 and recruit Cereblon as the E3 ubiquitin ligase, but they use different linkers. Cdk6 PROTAC 1 was kindly provided by Sarbjit Singh and Amarnath Natarajan (Eppley Institute for Cancer Research, University of Nebraska, Omaha, NE; Rana et al., 2019), Cdk6 PROTAC 2 was kindly provided by Yu Rao and Zimo Yan (School of Pharmaceutical Sciences, Tsinghua University, Beijing, China; Su et al., 2019).

#### Cdk6 activity assay

CCRF-CEM cells were collected and lysed in lysis buffer (PBS, 0.2% NP-40, 1 mM EDTA, protease inhibitor cocktail [Sigma-Aldrich] and 2 mM PMSF). Cells treated with 1 µM palbociclib (Sigma-Aldrich) for 24 h were used as a control. 60 µg total protein was plated into a 96-well Fluotrac 200 plate (Greiner) in triplicate. The assay was performed in 200 μl PBS supplemented with 5 mM MgCl_2_ and 0.5 mM ATP, and 200 nM Cdk6 peptide biosensor (Soamalala et al., 2021). Changes in fluorescence emission of the tetramethylrhodamine-labeled peptide biosensor were recorded at 30°C on a FLUOstar Omega (BMG; excitation 560 nm/emission 590 nm). Biosensor fluorescence was subtracted from the samples containing protein lysate, and relative fluorescence was calculated.

#### 200 kD cutoff ultrafiltration

5 × 10^7^ CCRF-CEM cells were collected per sample and lysed in lysis buffer (25 mM Tris-HCl, pH 7.5, 150 mM NaCl, 1 mM EDTA, 5% glycerol, 1% NP-40, 10 mM β-glycerophosphate) with a protease inhibitor cocktail (Sigma-Aldrich) and other inhibitors (1 mM PMSF, 10 mM NaF, 1 mM Na_3_VO_4_). 1 mM AMP (Acros Organics) or 10 mM citrate (Sigma-Aldrich) were also added to the lysis buffer as controls where indicated. Equal amounts of protein were filtered using the Disposable Ultrafiltration Units with molecular weight 200 kD cutoff (USY-20; Advantec MFS). 30 μg of total lysate or filtrate (<200 kD) were resolved by SDS-PAGE and immunoblotted with the indicated antibodies.

#### Cancer gene dependency scores

This study used DepMap 21Q3 dependency data for *FBXO7*, which had been generated from a CRISPR-Cas9 screen (available at https://DepMap.org; Tsherniak et al., 2017). Samples were grouped by their predefined lineage, and lineages with fewer than five cell lines were excluded from analyses. Final analyses included 1,020 cell lines. RNASeq expression analysis from Cancer Cell Line Encyclopedia (CCLE) was downloaded and analyzed from the https://DepMap.org database. A Kolmogorov Smirnoff significance test was done for Fbxo7 median expression for all cell lines against each sample’s specific expression.

#### Murine CD4^+^ T cell isolation and activation

Spleens were harvested from WT, heterozygous, or homozygous Fbxo7^LacZ^ mice and processed to a single cell suspension. For *n* = 2 of Fig. 3 E, cells were incubated in RBC lysis buffer (eBioscience) for 5 min prior to centrifugation. For all other data, viable lymphocytes were separated with mouse Lympholyte Cell Separation Media (Cedarlane Labs). CD4^+^ T cells were then isolated by negative selection using the MojoSort Mouse CD4 T Cell Isolation Kit (Biolegend). Isolated CD4^+^ T cells were seeded at 1 × 10^6^/ml in RPMI supplemented with 10% FBS, 100 U/ml penicillin and streptomycin, and 5 µM β-mercaptoethanol. To activate, cells were added to plates coated with 2 µg/ml α-CD3 (clone 145-2C11; Sigma-Aldrich) and containing 2 µg/ml soluble α-CD28 (clone 37.51; Biolegend) and incubated for the indicated duration. Cell viability and activation were measured by flow cytometry by staining with antibodies to CD4-PE (clone GK1.5; Invitrogen), CD25-PE/Cy7 (clone PC61; Biolegend), CD69-FITC (clone H1.2F3; Invitrogen), and an eFluor 780 fixable viability dye (eBioscience) for 30 min at 4°C in the dark. Samples were analyzed on a CytoFLEX S flow cytometer.

#### Seahorse extracellular flux analysis

Cells were harvested and resuspended in Seahorse XF RPMI medium (Agilent), supplemented with 2 mM glutamine (Thermo Fisher Scientific), 10 mM glucose (Thermo Fisher Scientific), and 1 mM pyruvate (Thermo Fisher Scientific), at pH 7.4. Cells were plated into a Seahorse XF96 Cell Culture Microplate (Agilent) precoated with 22.4 µg/ml Cell-Tak solution (Corning) at 3 × 10^5^ cells/well and incubated at 37°C for 45–60 min in a non-CO_2_ incubator. Cells were analyzed using the Seahorse XF Glycolytic Rate Assay Kit (Agilent) with 0.5 µM rotenone/antimycin A and 50 mM 2-deoxy-D-glucose. The assay was run at 37°C on a Seahorse XF96 analyser (Agilent) and data was analyzed using Wave software (Agilent).

#### Metabolite extraction

For metabolite profiling (Fig. 4 C and Fig. S3 C), murine CD4^+^ T cells were isolated and activated in a 12-well plate for 48 h. For ^13^C stable isotope tracing (Fig. 4 D), murine CD4^+^ T cells were isolated and activated in a 12-well plate with culture media containing 2 g/l *D*-Glucose-1,2-^13^C_2_ (Sigma-Aldrich) for 24 h. To extract metabolites, cells were harvested, washed twice in PBS, and resuspended in 200 μl ice cold metabolite extraction solution (50% liquid chromatography–mass spectrometry [LC-MS] grade methanol, 30% LC-MS grade acetonitrile, 20% ultrapure water, 5 μM valine-d8 as internal standard) per 1 × 10^6^ cells. Cells were incubated in a dry ice-methanol bath for 20 min, then at 4°C with shaking for 15 min. Samples were centrifuged at 13,000 rpm for 20 min, and the supernatant was collected into autosampler vials for LC-MS analysis.

#### Metabolite measurement by LC-MS

LC-MS chromatographic separation of metabolites was achieved using a Millipore Sequant ZIC-pHILIC analytical column (5 µm, 2.1 × 150 mm) equipped with a 2.1 × 20 mm guard column (both 5 mm particle size) with a binary solvent system. Solvent A was 20 mM ammonium carbonate, 0.05% ammonium hydroxide; Solvent B was acetonitrile. The column oven and autosampler tray were held at 40 and 4°C, respectively. The chromatographic gradient was run at a flow rate of 0.200 ml/min as follows: 0–2 min: 80% B; 2–17 min: linear gradient from 80% B to 20% B; 17–17.1 min: linear gradient from 20% B to 80% B; 17.1–22.5 min: hold at 80% B. Samples were randomized and analyzed with LC–MS in a blinded manner with an injection volume of 5 μl. Pooled samples were generated from an equal mixture of all individual samples and analyzed interspersed at regular intervals within sample sequence as a quality control. Metabolites were measured with a Thermo Fisher Scientific Q Exactive Hybrid Quadrupole-Orbitrap Mass spectrometer (HRMS) coupled to a Dionex Ultimate 3000 UHPLC. The mass spectrometer was operated in full-scan, polarity-switching mode, with the spray voltage set to +4.5 kV/−3.5 kV, the heated capillary held at 320°C, and the auxiliary gas heater held at 280°C. The sheath gas flow was set to 25 U, the auxiliary gas flow was set to 15 U, and the sweep gas flow was set to 0 U. HRMS data acquisition was performed in a range of *m/z* = 70–900, with the resolution set at 70,000, the automatic gain control target at 1 × 10^6^, and the maximum injection time at 120 ms. Metabolite identities were confirmed using two parameters: (1) precursor ion m/z was matched within 5 ppm of theoretical mass predicted by the chemical formula and (2) the retention time of metabolites was within 5% of the retention time of a purified standard run with the same chromatographic method.

#### Metabolomics data analysis

Chromatogram review and peak area integration were performed using the Thermo Fisher Scientific software Tracefinder (v.5.0). Correction for natural abundance was performed using the Accucor Package (v.0.2.3; Su et al., 2017) and the fractional enrichment was visualized using stacked bar graphs. For the total pools, the peak area for each detected metabolite was subjected to the “Filtering 80% Rule,” half-minimum missing value imputation, and normalized against the total ion count of the sample to correct any variations introduced from sample handling through instrument analysis. Sample were excluded after performing testing for outliers based on geometric distances of each point in the principal component analysis (PCA) score as part of the muma package (v.1.4; Gaude et al., 2013). Afterward, PCA was performed using the R base package stats (v.4.0.5; https://www.r-project.org/) with the function prcomp and visualised using the autoplot function of ggplot2 (v.3.3.3; Wickham, 2016) after loading the ggfortify package (v.0.4.11; Horikoshi and Tang, 2018; Tang et al., 2016). Differential metabolomics analysis was performed using the R package “gtools” (v.3.8.2; https://cran.r-project.org/web/packages/gtools/index.html) to calculate the Log_2_FC using the functions “foldchange” and “foldchange2logratio” (parameter base = 2). The corresponding P value was calculated using the R base package stats (v.4.0.5) with the function “*t*.test” (SIMPLIFY = F). Volcano plots were generated using the EnhancedVolcano package (v. 1.8.0; Blighe et al., 2021).

#### Quantitative RT-PCR

RNA extraction from murine CD4^+^ T cells was performed using the RNeasy Plus Mini Kit (Qiagen) and cDNA was generated using the QuantiTect Reverse Transcription Kit (Qiagen), both according to manufacturer’s instructions. Quantitative RT-PCR reactions were performed in triplicate using SYBR Green JumpStart Taq ReadyMix (Sigma-Aldrich). Primers used were: mouse Fbxo7 (forward 5′-CGC​AGC​CAA​AGT​GTA​CAA​AG-3′, reverse 3′-AGG​TTC​AGT​ACT​TGC​CGT​GTG-5′), mouse Cyclophilin (forward 5′-CCT​TGG​GCC​GCG​TCT​CCT​T-3′, reverse 3′-CAC​CCT​GGC​ACA​TGA​ATC​CTG-5′), mouse Actin (forward 5′-GAT​GTA​TGA​AGG​CTT​TGG​TC-3′, reverse 3′-TGT​GCA​CTT​TTA​TTG​GTC​TC-5′), and mouse Hprt (forward 5′-AGG​GAT​TTG​AAT​CAC​GTT​TG-3′, reverse 3′-TTT​ACT​GGC​AAC​ATC​AAC​AG-5′). Reactions were run on an Eppendorf Mastercycler ep realplex instrument and conditions were as follows: 95°C for 2 min; 45 cycles of 95°C for 15 s, 58°C for 20 s, 72°C for 15 s, 76°C for 8 s and read; followed by melting curve analysis from 65 to 95°C to confirm product specific amplification. Relative gene expression was calculated using the Pfaffl analysis method with normalization to three housekeeping genes (cyclophilin, actin, HPRT).

#### Cell cycle analysis

CCRF-CEM and MOLT-4 cells were incubated with 1 μM palbociclib for 24 h. Cells were harvested, washed in PBS, and slowly resuspended in ice-cold 70% ethanol by vortexing. Cells were fixed for at least 24 h, washed in PBS, and stained with propidium iodide (PI). Samples were analyzed on a Cytek DxP8 flow cytometer.

### Image capture, quantification, and statistical analysis

Immunoblots were captured on FujiFilm, and images captured and quantified using ImageJ processing software. Data are presented as mean ± SD. Statistical differences were calculated using Student’s two-tailed *t* tests with a significant cutoff of P < 0.05.

### Key resources table

Metabolomics Workbench datatrack_id:2912 study_id:ST001986 (Sud et al., 2016; http://dx.doi.org/10.21228/M8KM58).

### Resource availability

#### Lead contact

Further information and requests for resources and reagents should be directed to the lead contact, Prof. Heike Laman (hl316@cam.ac.uk).

#### Materials availability

Plasmids generated in this study have been deposited to Addgene (171786 and 171787). The Fbxo7^LacZ^ mouse line (Fbxo7^tm1a (EUCOMM)Hmgu^) is available from the International Mouse Phenotyping Consortium. Cell lines are commercially available.

## Online supplemental material

Fig. S1 shows the inhibition of cell cycle by palbociclib treatment and decrease in phosphorylation of pRb at Ser780. Fig. S2 shows the co-immunoprecipitation of Cdk6 and PFKP in MOLT-4 cells. Fig. S3 shows the Agilent Seahorse Glycolytic Rate Assay profiles for CCRF-CEM and Jurkat E6 cells with control or Fbxo7-targeting shRNA. In addition, the Chronos gene dependency scores for Fbxo7 from the DepMap portal and the expression of Fbxo7 in cell lines from the CCLE are shown. Fig. S4 shows data on comparisons of primary CD4^+^ T cells from WT and Fbxo7^LacZ/LacZ^ mice, including Agilent Seahorse Glycolytic Rate Assay profiles, PCA of metabolomics profiling after 48 h activation, and cell viability, with or without pyrimidines, after 24 h activation.

## Supplementary Material

SourceData F1 contains original blots for Fig. 1.

SourceData F2 contains original blots for Fig. 2.

SourceData F3 contains original blots for Fig. 3.

SourceData F5 contains original blots for Fig. 5.

SourceData FS1 contains original blots for Fig. S1.

SourceData FS2 contains original blots for Fig. S2.

## Data Availability

Metabolomics data have been deposited at MetaboLights and are publicly available as of the date of publication. Accession numbers are listed in the key resources table. CRISPR-Cas9 screening results for DepMap version 21Q3 are publicly available at https://DepMap.org (Tsherniak et al., 2017). Any additional information required to reanalyze the data reported in this paper is available from the lead contact upon request.
