# Supplementary material for: Fbxo7 promotes Cdk6 activity to inhibit PFKP and glycolysis in T cells
Source: J Cell Biol. 2022 Jun 7;221(7):e202203095. doi: 10.1083/jcb.202203095 (PMC9178409; doi:10.1083/jcb.202203095)
Supplement: SourceData F1 — contains original blots for Fig. 1. [file JCB_202203095_SourceDataF1.pdf]

Figure 1A

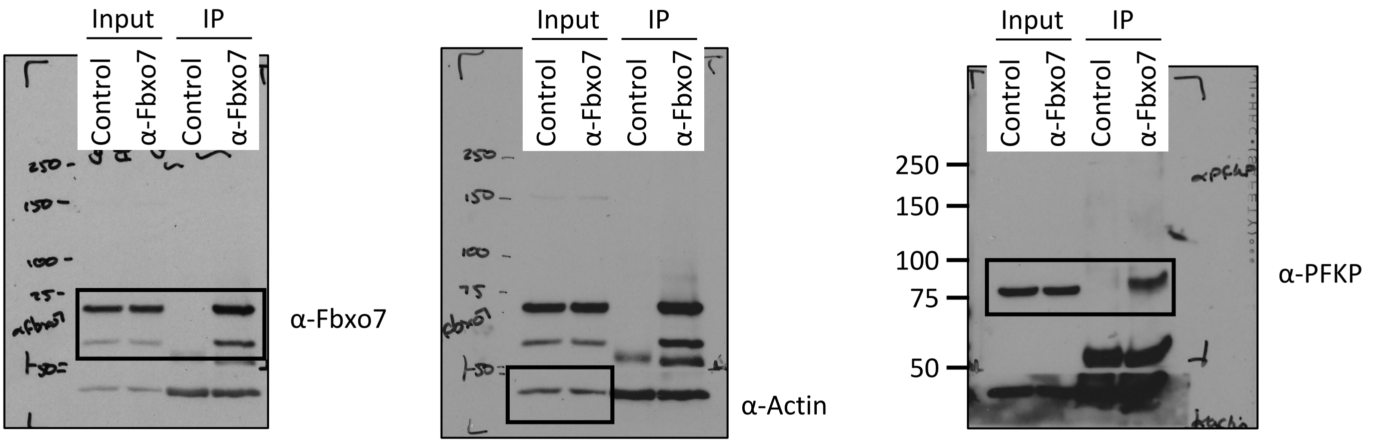

Figure 1C

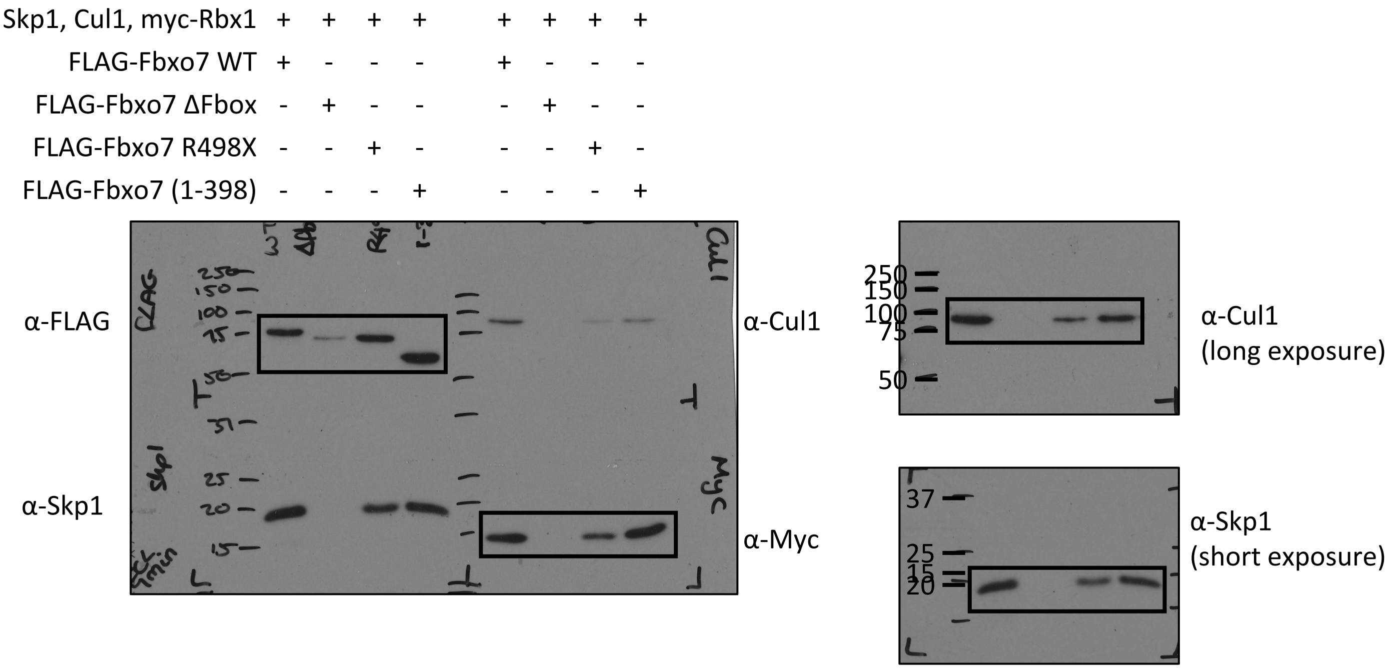

Figure 1D

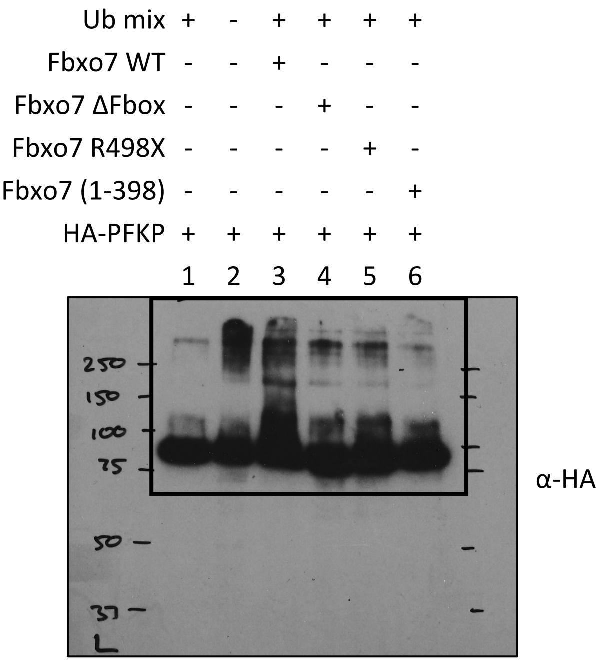

Figure 1E

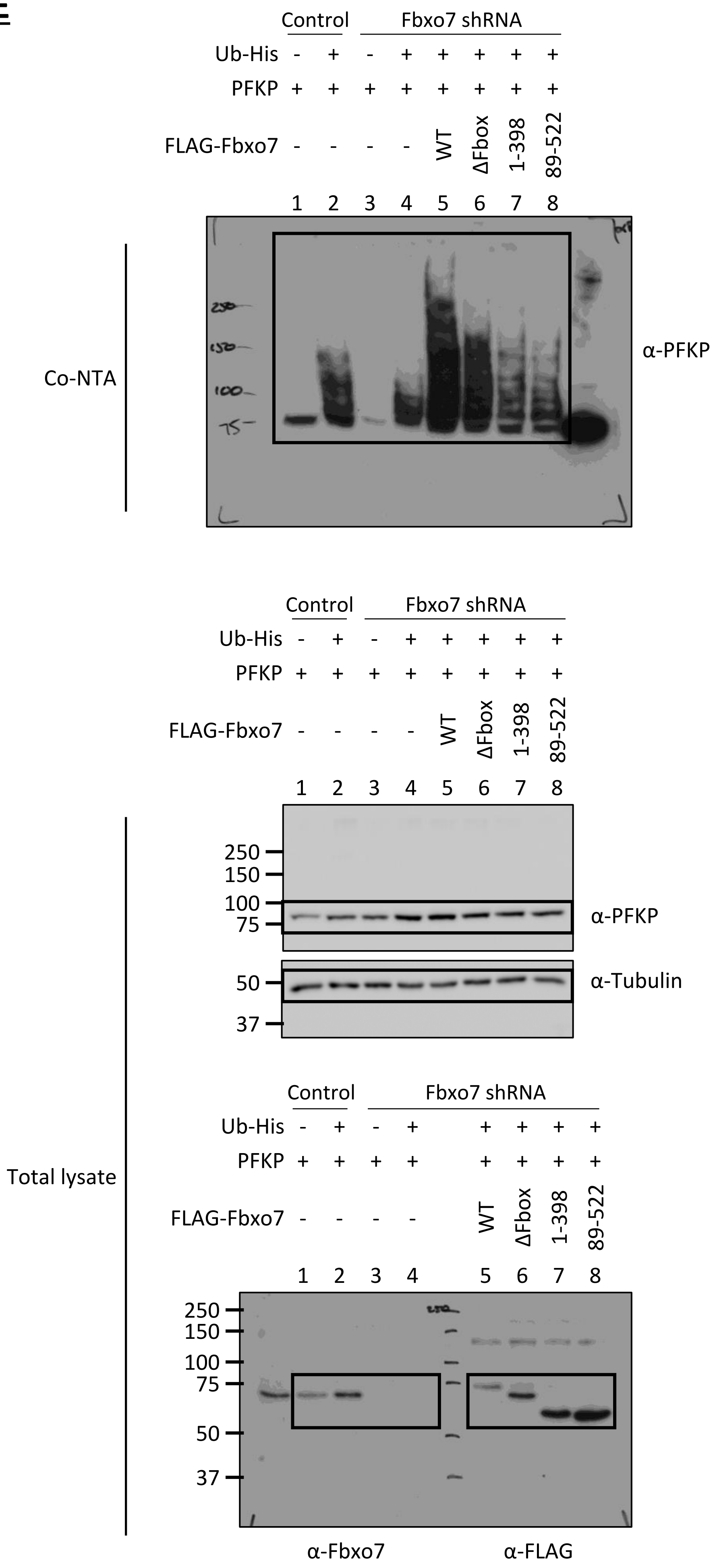
Control
Fbxo7 shRNA
Ub-His - + - + + + + +
PFKP + + + + + + + +
FLAG-Fbxo7 - - - - WT ΔFbox 1-398 89-522
1 2 3 4 5 6 7 8
Co-NTA
250
150
100
75
α-PFKP
Control
Fbxo7 shRNA
Ub-His - + - + + + + +
PFKP + + + + + + + +
FLAG-Fbxo7 - - - - WT ΔFbox 1-398 89-522
1 2 3 4 5 6 7 8
250
150
100
75
α-PFKP
50
37
α-Tubulin
Total lysate
Control
Fbxo7 shRNA
Ub-His - + - + + + + +
PFKP + + + + + + + +
FLAG-Fbxo7 - - - - WT ΔFbox 1-398 89-522
1 2 3 4 5 6 7 8
250
150
100
75
50
37
α-Fbxo7
α-FLAG
