# Supplementary figures and images for: Fbxo7 promotes Cdk6 activity to inhibit PFKP and glycolysis in T cells
Source: J Cell Biol. 2022 Jun 7;221(7):e202203095. doi: 10.1083/jcb.202203095 (PMC9178409; doi:10.1083/jcb.202203095)

Figure 2A

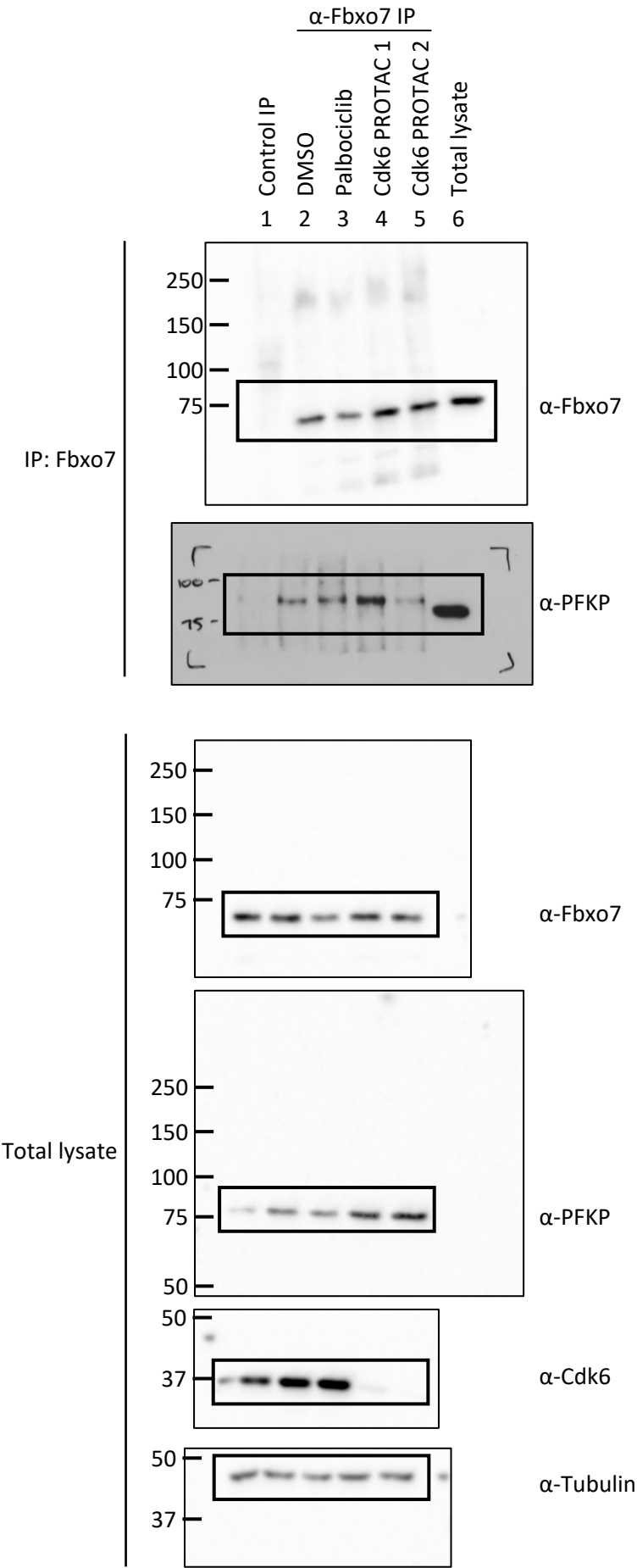

**Figure 2B**

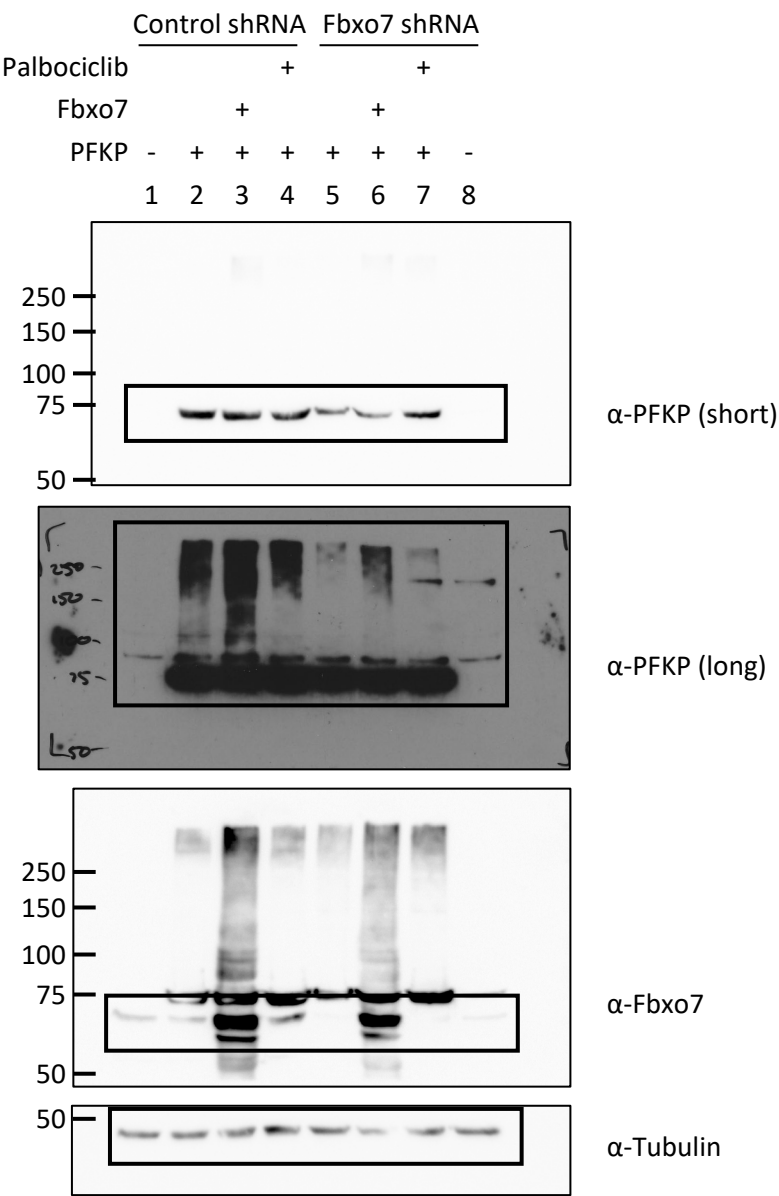

**Figure 2C**

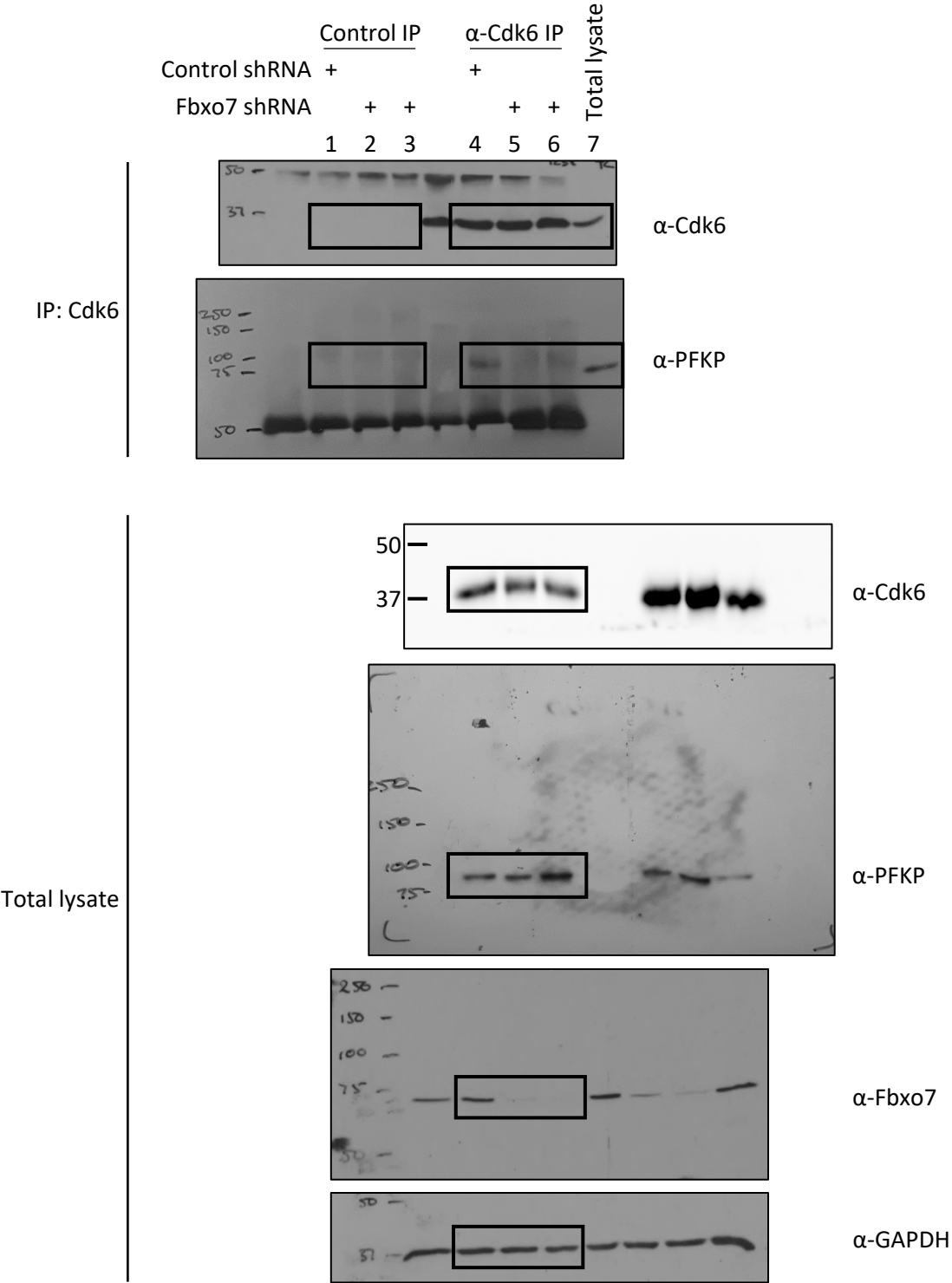

Supplement: SourceData F2 — contains original blots for Fig. 2. [file JCB_202203095_SourceDataF2.pdf]

Figure 3B

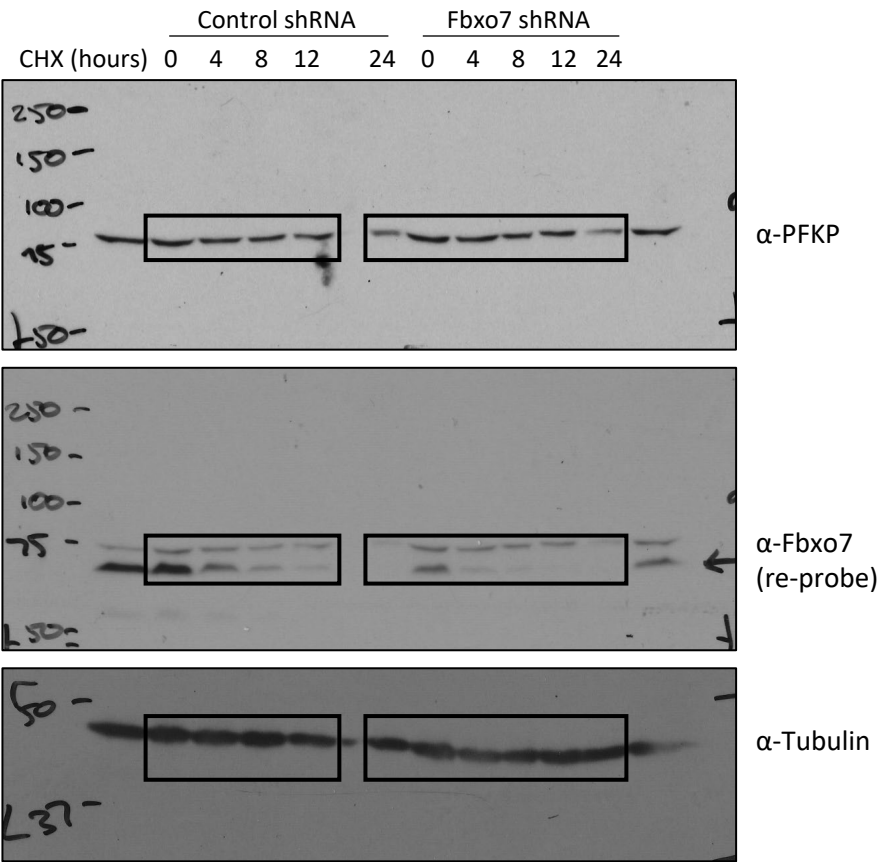

Figure 3D

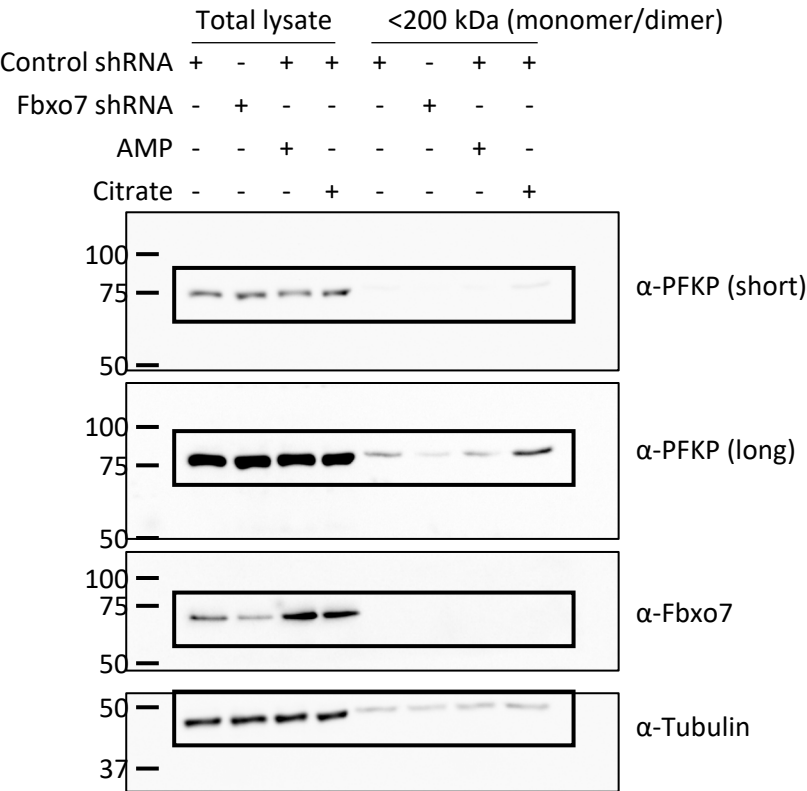

Supplement: SourceData F3 — contains original blots for Fig. 3. [file JCB_202203095_SourceDataF3.pdf]

**Figure 5B**

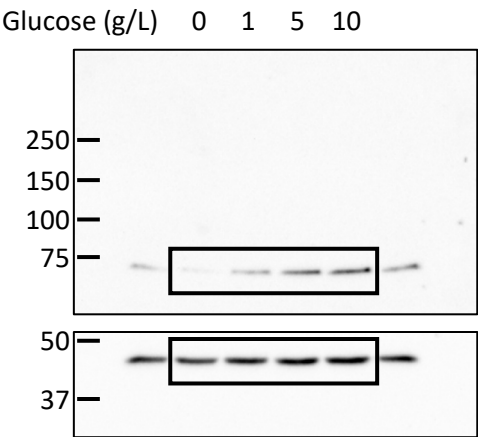

**Figure 5D**

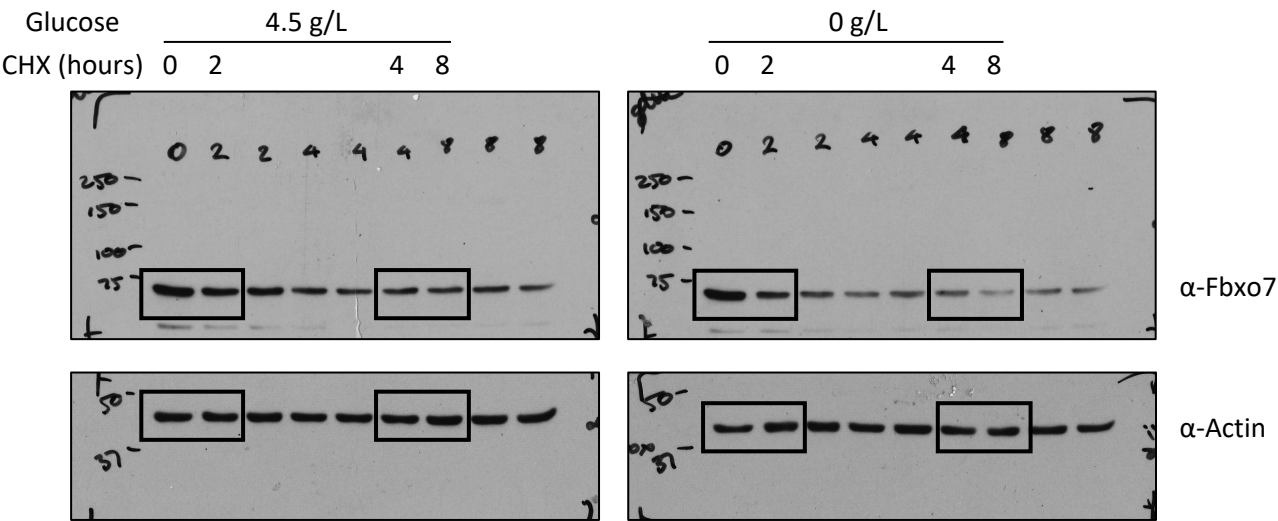

**Figure 5F**

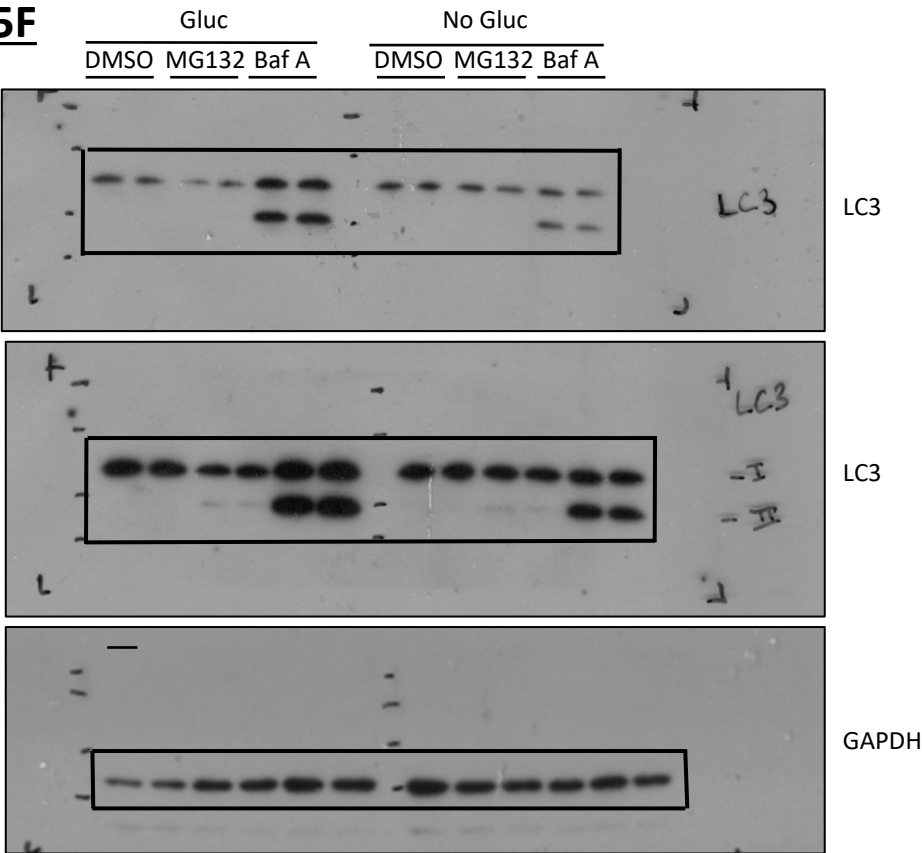

Supplement: SourceData F5 — contains original blots for Fig. 5. [file JCB_202203095_SourceDataF5.pdf]

**Supplementary Figure 1B**

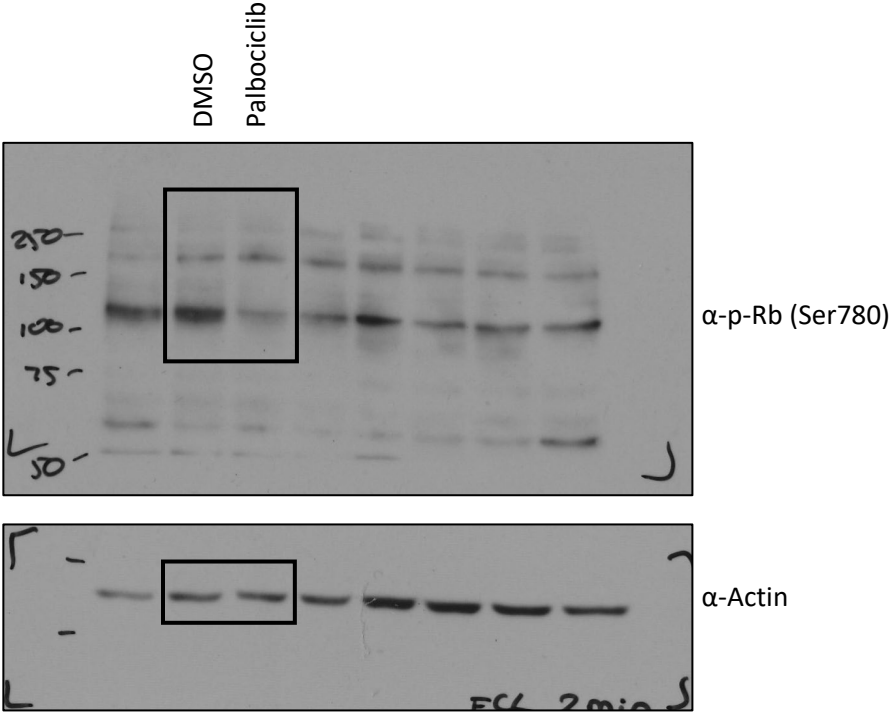

Supplement: SourceData FS1 — contains original blots for Fig. S1. [file JCB_202203095_SourceDataFS1.pdf]

Supplementary Figure 2

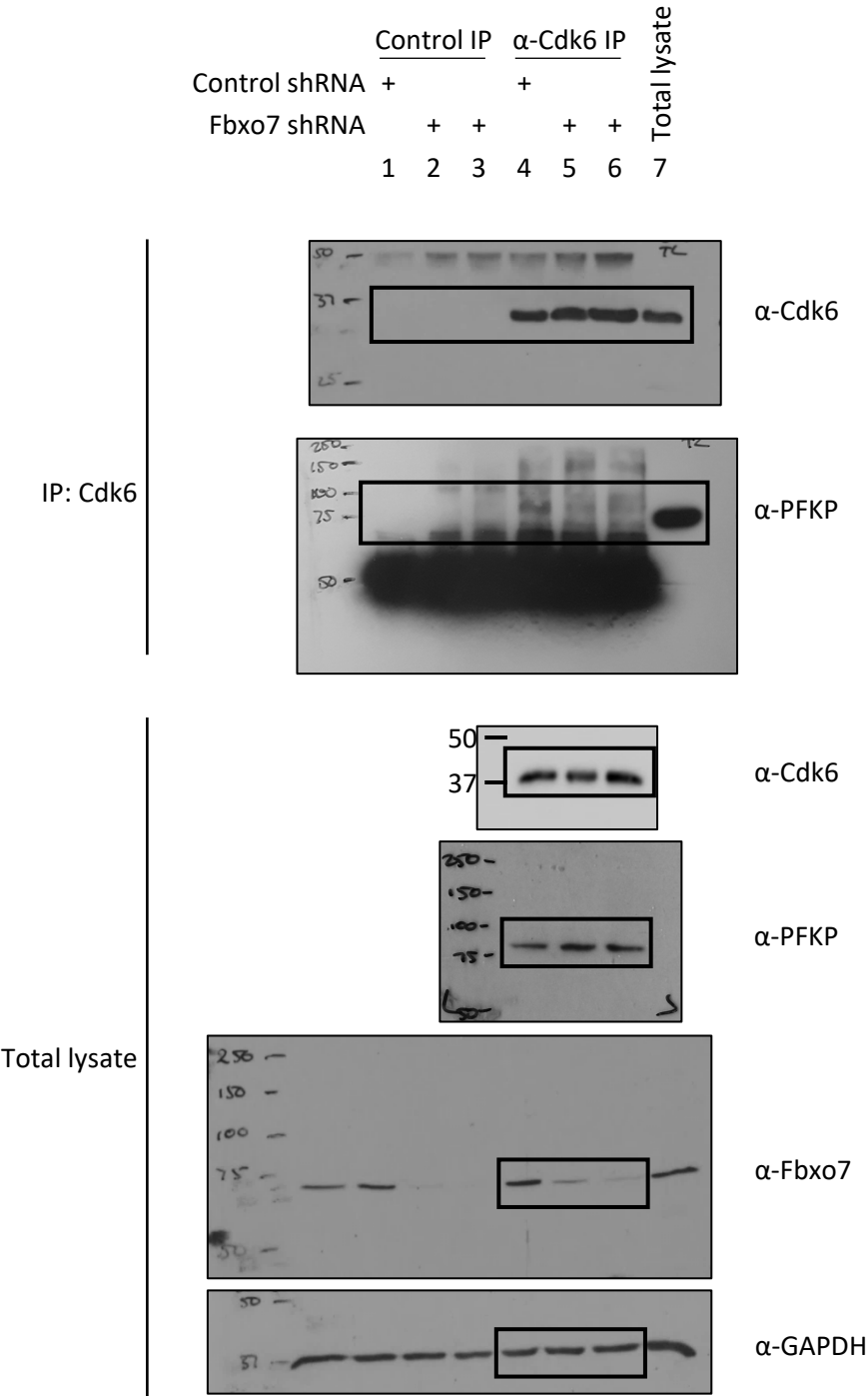

Supplement: SourceData FS2 — contains original blots for Fig. S2. [file JCB_202203095_SourceDataFS2.pdf]
